# Cognitive processes involved in smooth pursuit eye movements: behavioral evidence, neural substrate and clinical correlation

**DOI:** 10.3389/fnsys.2013.00004

**Published:** 2013-03-19

**Authors:** Kikuro Fukushima, Junko Fukushima, Tateo Warabi, Graham R. Barnes

**Affiliations:** ^1^Department of Neurology, Sapporo Yamanoue HospitalSapporo, Japan; ^2^Department of Physiology, Hokkaido University School of MedicineSapporo, Japan; ^3^Faculty of Health Sciences, Hokkaido UniversitySapporo, Japan; ^4^Faculty of Life Sciences, University of ManchesterManchester, UK

**Keywords:** smooth pursuit, eye movements, anticipation, efference copy, species comparisons, prediction, computational modeling, pathophysiology

## Abstract

Smooth-pursuit eye movements allow primates to track moving objects. Efficient pursuit requires appropriate target selection and predictive compensation for inherent processing delays. Prediction depends on expectation of future object motion, storage of motion information and use of extra-retinal mechanisms in addition to visual feedback. We present behavioral evidence of how cognitive processes are involved in predictive pursuit in normal humans and then describe neuronal responses in monkeys and behavioral responses in patients using a new technique to test these cognitive controls. The new technique examines the neural substrate of working memory and movement preparation for predictive pursuit by using a memory-based task in macaque monkeys trained to pursue (go) or not pursue (no-go) according to a go/no-go cue, in a direction based on memory of a previously presented visual motion display. Single-unit task-related neuronal activity was examined in medial superior temporal cortex (MST), supplementary eye fields (SEF), caudal frontal eye fields (FEF), cerebellar dorsal vermis lobules VI–VII, caudal fastigial nuclei (cFN), and floccular region. Neuronal activity reflecting working memory of visual motion direction and go/no-go selection was found predominantly in SEF, cerebellar dorsal vermis and cFN, whereas movement preparation related signals were found predominantly in caudal FEF and the same cerebellar areas. Chemical inactivation produced effects consistent with differences in signals represented in each area. When applied to patients with Parkinson's disease (PD), the task revealed deficits in movement preparation but not working memory. In contrast, patients with frontal cortical or cerebellar dysfunction had high error rates, suggesting impaired working memory. We show how neuronal activity may be explained by models of retinal and extra-retinal interaction in target selection and predictive control and thus aid understanding of underlying pathophysiology.

## Major cognitive influences on pursuit behavior

### Basic features of pursuit

#### Smooth pursuit initiation

**Figure 1:**
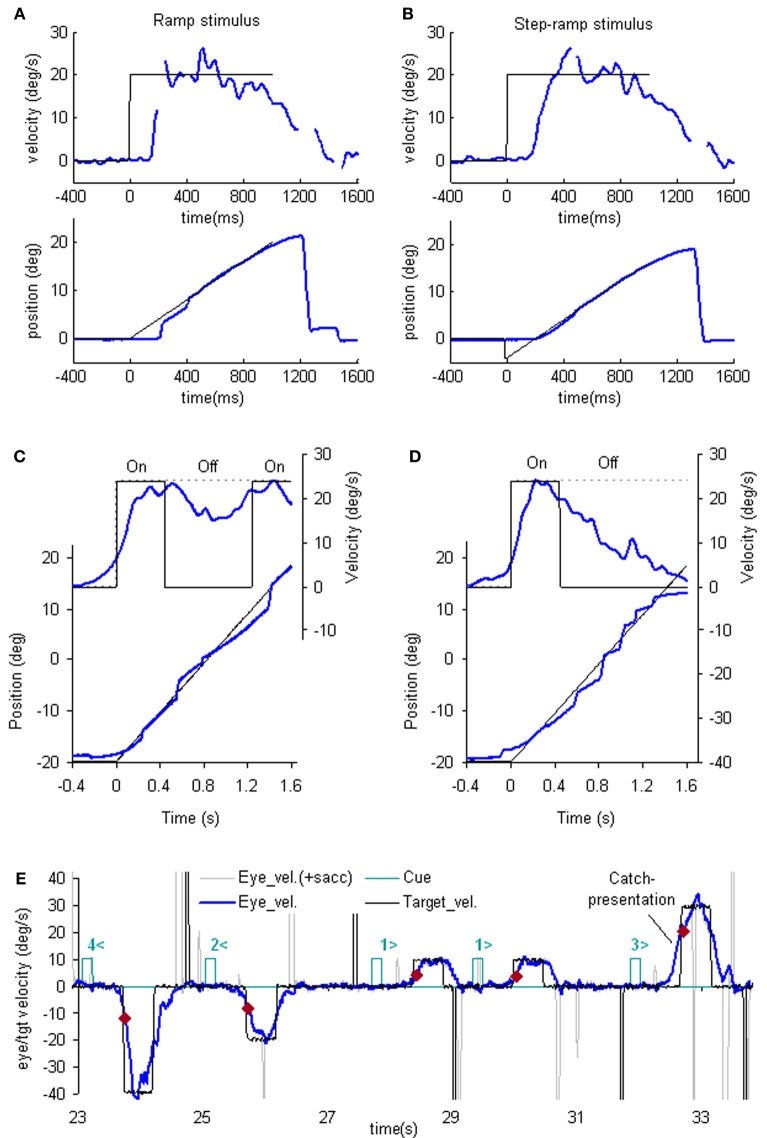
**Examples of reactive and anticipatory pursuit.** (**A** and **B**) Eye position (lower) and smooth eye velocity (upper) responses (blue traces) to ramp **(A)** and step-ramp **(B)** target motion stimuli (black traces) with velocity of 20 (blue)°/s. Gaps in smooth eye velocity traces represent locations of saccades removed in analysis. (**C** and **D**) Eye position (lower) and smooth eye velocity (upper) during target occlusion occurring 400 ms after target motion onset ( from Bennett and Barnes, 2003). In **(C)** target always reappears after 800 ms occlusion, whereas in **(D)** target never reappears but subject is instructed nevertheless to continue pursuit as if it will reappear. **(E)** Anticipatory smooth eye velocity responses made in response to target motion cues during presentation of randomized target velocities. Each cue comprised a digit (1, 2, 3, or 4) representing speed (10, 20, 30, or 40°/s, respectively) and a directional indicator (< or >) and occurred 600 ms before target onset. Anticipatory velocity markers (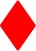) indicate eye velocity 50 ms after target onset (V50), prior to visual feedback. V50 increased as target speed increased (Jarrett and Barnes, 2005) as shown in these examples. In the catch presentation, target motion was unexpectedly delayed by 160 ms, resulting in earlier initiation of anticipatory movement and attainment of higher than normal V50.

The simplest way to assess pursuit performance is to examine the response to the sudden onset of an unexpected, constant velocity target motion (a ramp stimulus). Figure 1A shows typical human eye displacement responses to ramp stimuli of varying velocity; responses in the monkey are similar (Lisberger and Westbrook, 1985; Lisberger et al., 1987). In humans there is normally a latency of ~100–130 ms before smooth movements start (Tychsen and Lisberger, 1986; Carl and Gellman, 1987), whereas in the monkey shorter latencies of 80–100 ms are generally observed (Lisberger and Westbrook, 1985). The initial response delay results in a positional error that is corrected by a saccade that normally occurs after ~240 ms (Figure 1A) and realigns the image close to the fovea. Smooth eye displacement prior to the first saccade is often small but derivation of its velocity shows that the eye accelerates prior to the first saccade. However, after the saccade, eye velocity often jumps to a higher level (Lisberger, 1998) a feature referred to as post-saccadic enhancement. To eliminate the initial saccade or, at least, to ensure that it occurs later in the response, many investigators have used the so-called step-ramp stimulus (Rashbass, 1961), in which the target first jumps to one side, then makes a ramp in the opposite direction and crosses over the starting point in ~200 ms (Figure 1B). Eye movement normally starts somewhat later than for a simple ramp at ~130–150 ms after the step in humans (Rashbass, 1961). Once under way, the first 100 ms of the smooth response is effectively in an open-loop phase, since the delay in visual processing dictates that within this time period the retinal velocity error is not changed by the movement of the eye, as confirmed by open-loop studies (Carl and Gellman, 1987). Detailed examination of the step-ramp response has shown two distinct phases. In the initial 20–30 ms eye acceleration shows some increase with target velocity but not with starting position of the target motion (Lisberger and Westbrook, 1985; Tychsen and Lisberger, 1986), whereas, in the period 60–80 ms after onset there is a much greater modulation of eye acceleration by target velocity and a strong dependence on eccentricity of starting position. In humans, peak eye velocity is normally attained at a time that typically increases from ~220–330 ms after response onset as target velocity increases from 5 to 30°/s (Robinson et al., 1986).

#### Smooth pursuit maintenance

Following initial response onset eye velocity frequently overshoots target velocity and may oscillate at a frequency of 3–4 Hz in humans (Figure 1B) (Robinson et al., 1986). Oscillations normally die away within one or two cycles, although this varies between subjects and the size of the visual stimulus (Wyatt and Pola, 1987). With prolonged stimulation eye velocity settles to an average that is close to target velocity. Gain (the ratio of eye velocity to target velocity) is normally in the range 0.9–1.0 for target velocities <20°/s. Meyer et al. (1985) showed that gain in humans could remain as high as 0.9 up to ~90°/s, but declines at higher velocity. If gain falls substantially below unity, corrective saccades are made to realign the target image on the fovea.

#### Smooth pursuit termination

Ocular pursuit is an example of a negative feedback control system and if it were linear, the response evoked by termination of a ramp stimulus should be the inverse of that at initiation; eye velocity should thus oscillate when reaching zero velocity (i.e., in the transition from pursuit to fixation). However, when target motion ceases unexpectedly, following a latency of ~100 ms, eye velocity generally decays to zero with a time constant of ~100 ms (Robinson et al., 1986; Pola and Wyatt, 1997) without evidence of overshoot. This was taken as evidence that fixation does not represent pursuit at zero velocity; rather, the simple decay of eye velocity was thought to represent the disengagement of pursuit (Robinson et al., 1986). As discussed later the response at termination actually depends on the subject's expectation.

### The role of retinal and extra-retinal mechanisms

**Figure 2:**
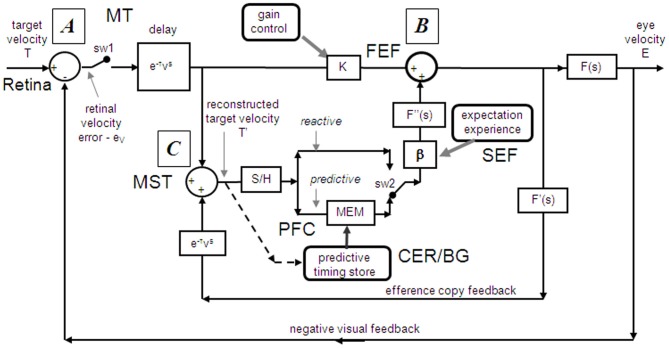
**Model of ocular pursuit.** The basis of the model is a negative feedback loop in which retinal velocity error is processed by internal dynamics F(s) with variable gain K and a delay (τ_v_) of ~80–100 ms. The negative visual feedback is supplemented by extra-retinal input from either a *reactive* or *predictive* loop. The input to both reactive and predictive pathways comes from sampling (for ~150 ms) and holding a copy of the reconstructed target velocity signal (T′) in module S/H. The reactive loop can thus sustain eye velocity even if visual input is withdrawn (i.e., if sw1 is opened). The predictive loop includes a more robust short-term memory (MEM), which can hold velocity information over longer periods and during fixation. Both direct and indirect pathways feed out through an expectation-modulated gain control (β < 1) and filter F”(s). In a reactive response, S/H output is fed out directly but is also temporarily stored in MEM. In predictive mode, output of MEM is fed out to form an anticipatory response with timing based on external cues or on the detection of direction changes in the reconstructed target velocity signal and held in the predictive timing store. F″(s) = F′(s) = F(s) = (1 + Te.s)^−1^. Te = 0.12 s. Non-linear gain function approximated by: K = K_0_ (1 + e/e_0_)^−0.5^, where e = retinal error, e_0_ = 4°/s, typically, K_0_ ≈ 2.4. For information on putative brain areas (MT, MST, FEF, SEF, PFC, CER, and BG) see section “Allocation of Model Functions to Specific Brain Areas.” Adapted from Barnes and Collins (2011).

Models based on control theory have been used very successfully to describe the dynamic characteristics of pursuit (Robinson et al., 1986). The major problem lies in simulating the relatively rapid rise of eye velocity combined with the high levels of closed-loop gain normally attained. These two requirements cannot be met by simple negative feedback without the system exhibiting unstable oscillation because of the time delays associated with visual motion processing; although some oscillation is observed (Figure 1), it is generally of small amplitude. The most widely accepted way in which stability is thought to be achieved is through the positive feedback of an efference copy of eye movement, as represented by the *reactive* loop of the model shown in Figure 2, a proposal originally made by Yasui and Young (1975). Elaborations of this concept have formed the basis for a number of subsequent models (Robinson et al., 1986; Krauzlis and Lisberger, 1994; Deno et al., 1995; Krauzlis and Miles, 1996).

An important generic feature of these models is that if visual feedback is suddenly cut off, the efference copy feedback loop can sustain the response to some extent (Figure 1C). In effect, the loop acts as a simple, but volatile, velocity memory. This fits with an important observation, that during pursuit of a target that unexpectedly disappears, smooth eye movements do not simply stop but can be sustained, albeit at reduced velocity, in both humans (Von Noorden and Mackensen, 1962; Becker and Fuchs, 1985) and monkeys (Newsome et al., 1988). This occlusion paradigm has been used frequently to reveal features of the internal (or extra-retinal) drive mechanisms for pursuit.

Recent evidence has called into question the validity of this simple efference copy model (Barnes and Collins, 2008a,b). Although target occlusion experiments lead to a decrease in eye velocity, there is often a recovery of eye velocity (Figure 1C) prior to expected target reappearance (Becker and Fuchs, 1985; Bennett and Barnes, 2003) that cannot be easily explained by such models. Moreover, eye velocity can increase above the level attained prior to disappearance if target velocity is expected to increase at the end of the occlusion (Barnes and Schmid, 2002; Bennett and Barnes, 2004). Also, corrective saccades during occlusion tend to align eye position with the expected target trajectory (Bennett and Barnes, 2003), as shown in Figure 1D (see section “The Role of Expectation and Mismatch Detection in Predictive Pursuit” for details), suggesting that true velocity has been retained and integrated to estimate future target position despite smooth eye velocity reduction (see also Orban de Xivry et al., 2008, 2009). Related evidence for such positional corrections has been obtained in monkey (Barborica and Ferrera, 2003). This suggests that initial target velocity is sampled and stored in a less volatile form of memory than implied by continuous efference copy feedback.

### The role of expectation and mismatch detection in predictive pursuit

One of the problems in assessing the validity of the efference copy idea is that it is difficult to demonstrate the existence of internally driven eye movements in the absence of vision unless there has been some prior visual input (as in Figure 1C). In particular, it is difficult to initiate smooth eye movements in the absence of visual input. Early experiments suggested a capacity to evoke only very low velocity smooth pursuit at will (Heywood, 1972; Kowler and Steinman, 1979), but subsequent experiments have revealed that much higher velocities can be evoked as anticipatory movements during repeated stimulation in humans (Becker and Fuchs, 1985; Barnes et al., 1987; Boman and Hotson, 1988) and monkeys (Ilg, 2003; Missal and Heinen, 2004). In addition, Jarrett and Barnes (2001, 2002) have shown that subjects can use symbolic cues that reliably indicate the speed and direction of an upcoming target motion to generate appropriately scaled and directed anticipatory movements, even when target movements are randomized in speed and direction (Figure 1E). More surprisingly, smooth movements can be evoked in the absence of any retinal slip, e.g., when following a series of target steps (Barnes et al., 1987; Barnes and Asselman, 1992) or when shifting attention to a more eccentric location on an image that is stabilized on the fovea. The latter generates smooth movement scaled to the eccentricity and in the direction of the shift (Grüsser, 1986; Sheliga et al., 1994; Barnes et al., 1995). These findings suggest a more generalized mechanism for generating smooth pursuit when the target is expected to move from one position to another. In all cases, though, expectation is the critical factor that allows initiation of such internally generated movements (Kowler, 1989; Barnes et al., 2002). Expectation is also a critical factor in the maintenance of eye velocity during occlusion; without expectation of target reappearance, eye velocity rapidly declines toward zero (Mitrani and Dimitrov, 1978; Bennett and Barnes, 2004), even when the subject attempts to continue pursuit, as evidenced by the fairly successful ability to follow the future target movement (Figure 1D).

The dependence on expectation is probably associated with the need to detect any mismatch between prediction and sensory feedback. Such a mechanism is essential if false predictions generated by extra-retinal mechanisms are to be rectified. Effects of expectation can be readily revealed by catch trials in which unexpected stimulus changes occur (see example in Figure 1E). In general, inappropriate prediction occurs for at least 100 ms after expected target appearance, i.e., the expected latency of visual feedback (Barnes and Asselman, 1991; Barnes et al., 2000). Absence of conflict is probably the factor that allows smooth pursuit to be continued when the image is stabilized on the retina (Cushman et al., 1984; Barnes et al., 1995).

### Evidence of sampling and storage in the initial pursuit response

**Figure 3:**
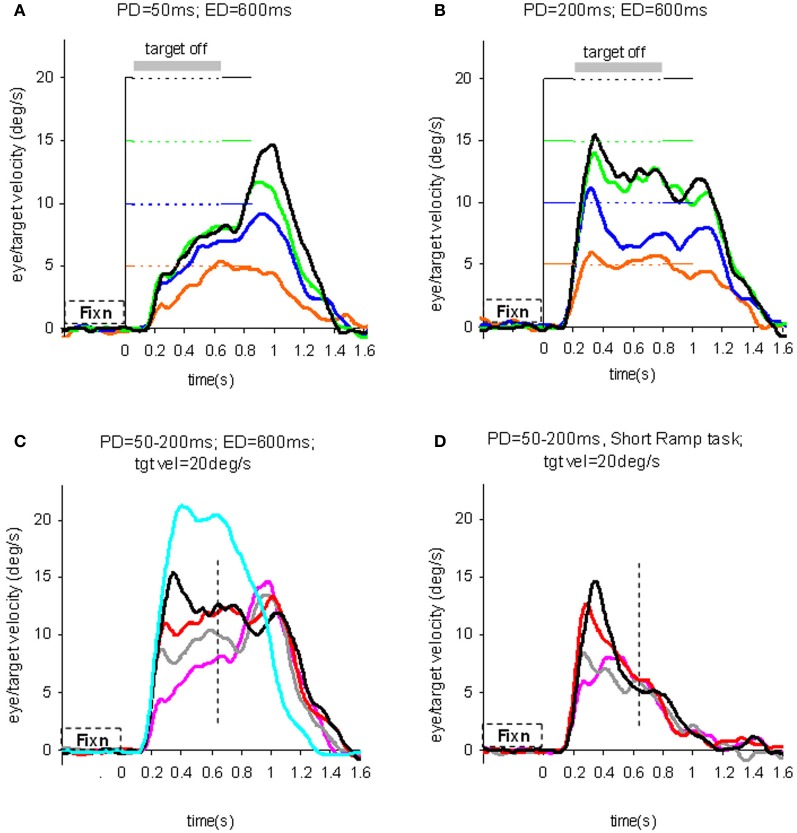
**Eye velocity trajectories during target extinction.** Smooth eye velocity averaged across all six subjects in the Mid-ramp Extinction **(A–C)** and Short Ramp **(D)** tasks. In **(A)** and **(B)** target velocity = 5°/s (orange), 10°/s (blue), 15°/s (green), or 20°/s (black); PD = 50 ms in **(A)**, PD = 200 ms in **(B)**. In **(C)** and **(D)** target velocity = 20°/s; PD = 50 ms (magenta), 100 ms (gray), 150 ms (red), or 200 ms (black). Also shown in **(C)** is the average smooth eye velocity in the Control response for target velocity = 20°/s (cyan trace). In **(C)** and **(D)** dotted lines denote 650 ms after target onset; note that for these examples target extinction occurred at a different time for each data series. PD = initial target exposure duration; ED = duration of target extinction. From Barnes and Collins (2008a).

To test the hypothesis that target velocity might be sampled at the onset of the pursuit stimulus, Barnes and Collins (2008a) devised an experiment in which the target was presented for a very brief period (*PD* = 50–200 ms) at the beginning of the ramp and was then extinguished for a period (ED) up to 600 ms. Crucially, the direction, speed, initial presentation duration and time of initiation of the ramp were randomized with the objective of determining whether subjects were able to extract motion information within the brief presentation so as to scale their smooth eye velocity to target velocity during occlusion. Various sources of behavioral (Lisberger, 1998; Bennett et al., 2007), and neurophysiological evidence (Osborne et al., 2004) suggested that 200 ms should be sufficient to fully extract target velocity information. Given the brief duration of presentation, the retinal component of pursuit was expected to be considerably reduced, allowing any extra-retinal component to be clearly identified. As shown in (Figures 3A–C), there were two distinct phases of the response to this Mid-ramp extinction condition, an initial rapid increase in eye velocity followed by a secondary, more sustained response. The initial component closely followed the response in a control condition (cyan trace, Figure 3C) in which the target was continuously visible, but this initial component reached a peak that increased as the duration of target presentation (PD) increased; this represents the visually-driven component of the pursuit response. The secondary component, however, which continued well after target extinction, represents the extra-retinal component of pursuit. For the shortest presentations (50 and 100 ms) the secondary component continued to increase beyond the initial peak whereas for the 200 ms presentation there was a decline from the initial peak toward an asymptotic level which was similar for 150 and 200 ms. Importantly, this asymptotic level increased as target velocity increased (Figures 3A,B).

**Figure 4:**
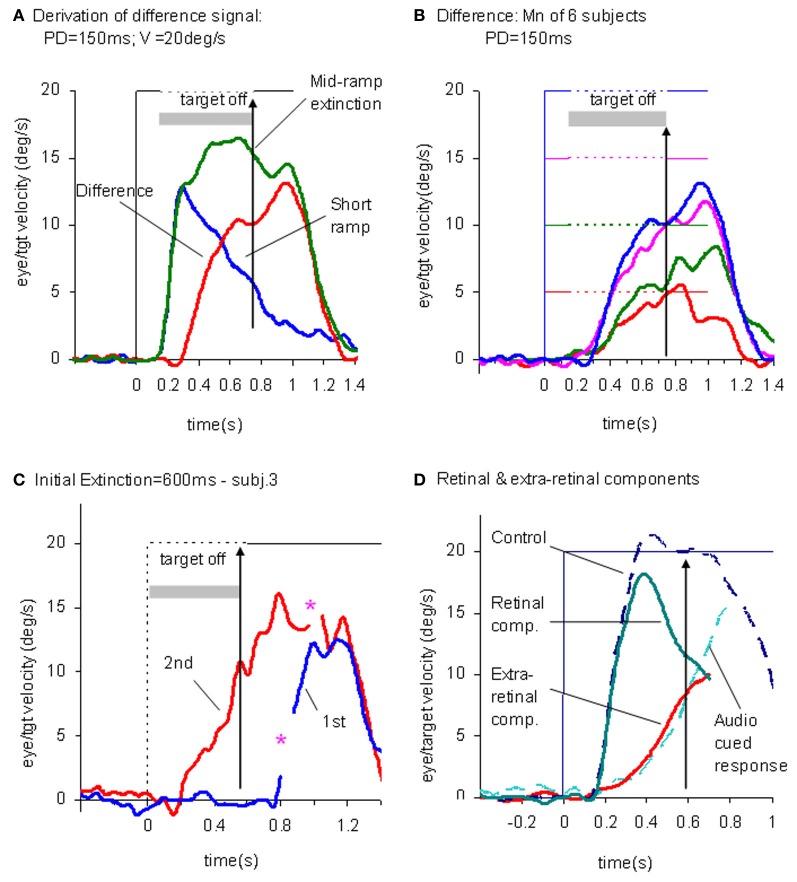
**Derivation of retinal and extra-retinal pursuit components. (A)** Comparison of average eye velocity in the Mid-ramp Extinction (green trace), and Short Ramp (blue trace) conditions. The red trace represents the difference between these conditions. Target velocity = 20°/s; Initial target exposure duration (PD) = 150 ms. **(B)** The difference signal averaged across all six subjects for PD = 150 ms for each target velocity [5 (red), 10 (green), 15 (magenta), and 20°/s (blue)]. Gray shading indicates period of target extinction. **(C)**. Example response from single subject during first (blue trace) and second presentations (red trace) of the Initial Extinction condition. Asterisks indicate occurrence of saccades. **(D)** Predicted behavior of retinal and extra-retinal components of pursuit during initial response to ramp target motion at 20°/s. Extra-retinal component (red trace) is derived from average Initial Extinction responses of six subjects, but terminates 80 ms after target onset. Cyan trace shows data obtained from similar Initial Occlusion experiment (Collins and Barnes, 2006, mean of 16 and 24°/s responses) in which response is aligned to audio cue occurring 700 ms before target appearance. Data in **(A–C)** derived from Barnes and Collins (2008a,b).

This experiment also took advantage of the finding that the continuation of the extra-retinal component would be dependent on the expectation of target reappearance by comparing the Mid-ramp extinction condition with a Short Ramp condition in which the target failed to reappear. It was argued that subtraction of the Short-ramp response (Figure 3D) from the Mid-ramp response (see Figure 4A) should give an indication of the temporal development of the expectation-dependent extra-retinal component. As shown in Figure 4A, because the initial visually-driven components of Mid-ramp and Short-ramp responses were very similar, their effect was eliminated, revealing that the difference signal increased with time with much lower acceleration than the visually-driven component. Importantly, eye velocity at the end of occlusion increased with target velocity (Figure 4B), thus providing evidence that target velocity had been sampled during the initial presentation and held as a reference level in a form of working memory.

### Similarity of extra-retinal pursuit component and anticipatory smooth pursuit

In an attempt to determine how the extra-retinal component might develop in the complete absence of initial retinal input a further experiment was devised (Barnes and Collins, 2008b). Subjects initially fixated a stationary target for a randomized period of 500–1000 ms. The target was then extinguished for 600 ms but the subject was informed that target extinction indicated that its unseen motion had started; thus, when it reappeared, the target was already in an eccentric position and in motion. This paradigm was referred to as the Initial Extinction condition. Since target velocity was unknown at the start of motion, the stimulus was presented in blocks of repeated, identical stimuli, but target speed and direction were randomized between blocks. In the first presentation of each block the response started ~100 ms after target appearance whereas in the second and subsequent presentations anticipatory smooth movements were made during the initial occlusion (Figure 4C). These anticipatory responses were initiated with a mean latency of 196 ms after the offset of fixation, i.e., ~50–60 ms after the onset of the visually-driven response, and once initiated, exhibited a relatively slow build-up of eye velocity during the remaining occlusion. Eye velocity at the end of occlusion increased significantly with target velocity, in line with previous observations relating to anticipatory eye movements (Collins and Barnes, 2006). These anticipatory responses could not be distinguished from the difference signal (Figure 4B) described above, for the same subjects and target velocities (Barnes and Collins, 2008b). Furthermore, in an attempt to mimic conditions that are more representative of the underlying processes in the Mid-ramp condition, a modified technique was subsequently developed, in which the initial velocity estimate had to be obtained from a single Short-ramp presentation [i.e., a brief sample (150 ms) of target motion]. This was followed by a period of fixation prior to presentation of a single Initial Extinction condition (Ackerley and Barnes, 2011). This method yielded very similar anticipatory responses in the Initial Extinction condition. This study was conducted in both head-fixed and head-free pursuit conditions; it demonstrated that subjects are able to store target motion information in each Short-ramp presentation and use it to initiate appropriately scaled anticipatory movements of both head and gaze in the Initial Extinction condition.

The picture that develops from these findings is that when the subject attempts to follow a randomized ramp stimulus the retinal and extra-retinal components operate as shown in Figure 4D. The retinal component has a latency of ~100–130 ms, but when initiated, has relatively high acceleration and allows eye velocity to reach target velocity in 200–300 ms. The underlying extra-retinal component starts ~50 ms later and develops more slowly, probably taking around 500–600 ms to reach peak velocity. Evidence suggests that it is a much noisier estimate of target velocity than that provided by visual feedback (Ackerley and Barnes, 2011). As the extra-retinal component develops it gradually takes over from the retinal component, which then diminishes toward zero as a natural consequence of its dependency on the rapidly decreasing retinal velocity error. The extra-retinal component is not a trivial proportion of the total response; it can reach gains >0.6 prior to target appearance [see cyan trace in Figure 4D; data from Collins and Barnes (2006)]. Importantly, this does not mean that the retinal component is eliminated; it still remains active in most circumstances and can correct for unexpected changes in the stimulus. Hence, when transient target motion probes are used during steady state pursuit the expected reactive response is still evoked (Schwartz and Lisberger, 1994).

### Target selection and gain control

When humans are confronted with multiple moving stimuli (e.g., a typical street scene) they must select which particular moving object to pursue. One way to accomplish this would be to enhance the visual feedback of the selected object in relation to other stimuli by increasing the open-loop gain (K in the model, Figure 2) associated with that target. Evidence for such gain increases comes from experiments in which clear differences have been shown in the magnitude of responses evoked by active pursuit as opposed to passive stimulation in which the subject simply stares at the moving target (Barnes and Hill, 1984; Barnes and Crombie, 1985; Pola and Wyatt, 1985). It has also been shown that when a high frequency (e.g., 5 Hz) single cycle perturbation is superimposed on constant velocity target motion the eye velocity gain associated with the perturbation increases with target velocity in both monkey (Schwartz and Lisberger, 1994) and man (Churchland and Lisberger, 2002). Once the pursuit target has been selected and the eye moves across the remaining non-selected stimuli, the passive response induced should reduce pursuit velocity. Such interactions can be demonstrated for pursuit against large backgrounds, although the decrease is normally no more than 10–20% (Yee et al., 1983; Collewijn and Tamminga, 1984; Kowler et al., 1984; Barnes and Crombie, 1985; Worfolk and Barnes, 1992; Kasahara et al., 2006). Although this type of interaction explains some behavior in the steady state, there are clearly other mechanisms at play (Keller and Khan, 1986; Kimmig et al., 1992; Mohrmann and Thier, 1995).

Surprisingly, even quite small targets (or distracters) can have a passive influence on smooth pursuit (Cheng and Outerbridge, 1975; Barnes and Hill, 1984). When a simple distracter is presented simultaneously with a pursuit target an attention-modulated selection process occurs before pursuit initiation. Ferrera and Lisberger (1995, 1997) showed that the initial open-loop response is a vector average of the responses that would be made to individual stimuli. After an initial period (~50 ms) a saccade is made to one of the targets and post-saccadic eye velocity is then in the direction of the selected target. If the distracter moves in the opposite direction or is stationary an increase in latency alone is observed (Lisberger and Ferrera, 1997; Knox and Bekkour, 2004). In general, changes in pursuit gain observed in the presence of backgrounds or distracters result from physical characteristics such as size and peripheral location of competing stimuli, but perhaps most importantly, by the influence of attention (Kerzel et al., 2008), which raises the gain for the selected target and/or suppresses the gain for competing stimuli.

### Updating the pursuit model

If as we propose, the extra-retinal component underlying the maintenance phase is produced by the same mechanism as anticipatory pursuit it is necessary to suggest how this might be incorporated in a more general model of pursuit. This requires additional features to be added to the efference copy model, notably the inclusion of a second internal loop, the *predictive* pathway (Figure 2). Whereas the *reactive* pathway is assumed to function during randomized responses and generates an extra-retinal component scaled to the initial target velocity as shown in Figure 4, the *predictive* pathway holds velocity samples captured during prior stimulation in a form of working memory (MEM). This *predictive* pathway enables motion information to be retained during fixation and thereby allows appropriately scaled anticipatory movements to be released in advance of future eye movement, given appropriate expectation of target appearance. The results of numerous experiments (Barnes and Asselman, 1991; Kao and Morrow, 1994; Barnes and Donelan, 1999) have shown that anticipatory movements evoked by repeated motion stimuli have a velocity that is scaled in proportion to target velocity, even when the subject fixates a stationary target and simply views but does not pursue the initial presentation (Barnes et al., 1997, 2000; Burke and Barnes, 2008a). This implies that target speed information can be stored independently of ongoing eye movement, a feature that can be accomplished by assuming that the target velocity estimate is internally reconstructed by summation of efference copy and retinal error independently of the main oculomotor drive, as shown in Figure 2 (junction C). Results of experiments using complex motion stimuli comprising sequences of ramps with randomized speed and direction (Barnes and Schmid, 2002; Collins and Barnes, 2005; Burke and Barnes, 2007) have shown that multiple levels of velocity may be retained within MEM. The output of variable levels of stored information from MEM over time may constitute a basis for the dynamic representation of target motion described by Orban de Xivry et al. (2008).

If stored motion information is to be used effectively for prediction it needs to be released at an appropriate time to minimize velocity error. The release of the output from MEM is dependent on timing that can be derived from external cues (Boman and Hotson, 1988; Barnes and Donelan, 1999; Jarrett and Barnes, 2005) or cues derived from the motion itself if it is periodic (Barnes and Asselman, 1991). Timing is of importance not only for response initiation but also for its termination. Even for a simple ramp stimulus of known duration there is a tendency to reduce eye velocity in anticipation of the ramp termination (Robinson et al., 1986; Kowler and McKee, 1987; Boman and Hotson, 1988) as shown by the control examples in Figure 4. Krauzlis and Miles (1996) showed that the dynamics of pursuit offset are significantly affected by the subject's experience. When ramp stimuli of identical duration are repeated, timing becomes pre-programmed, so that an unexpected increase in duration results in inappropriate eye velocity reduction for ~400 ms (Barnes et al., 2005).

An important feature of the model (Figure 2) is that output from the *reactive* and *predictive* loops is gated by expectation, which is represented by the variable gain β (≤ 1). This includes a mechanism for detecting mismatch between the predictive velocity and available visual feedback. This reflects the fact that, in anticipatory mode, the system has changed from being one that relies on visual feedback to one that generates a predictive estimate of the required motor drive and uses feedback to check that this is correct. Importantly, it would not be possible for the *reactive* and *predictive* pathways to operate simultaneously since this would overestimate target velocity, so it must be assumed that activation of the *predictive* pathway automatically leads to inhibition of the *reactive* pathway. This model has been used to provide a very effective simulation of responses in the Mid-ramp, Short ramp, Initial Extinction and Control conditions (Barnes and Collins, 2008b). It should also be noted that, by incorporating two working memory components, one that holds current motion information (S/H) and another that holds prior information MEM, this model provides the necessary structures for motion perception tasks in which current and prior motion stimuli are compared (Greenlee et al., 1995).

## Neural substrate of working memory and movement preparation for smooth-pursuit

### Major pathways related to smooth-pursuit eye movements

**Figure 5:**
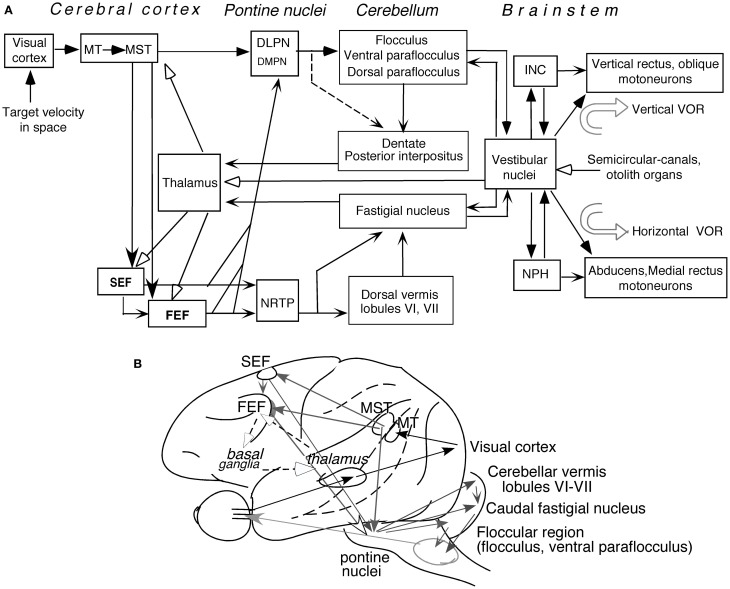
**Major pathways related to smooth-pursuit eye movements.** Major pathways related to smooth-pursuit **(A,B)**. Open arrowheads with dashed lines in **(B)** schematically indicate a proposed smooth-pursuit efference copy loop between the caudal FEF and the basal ganglia through the thalamus which is not shown in **(A)** (adapted from Cui et al., 2003). Abbreviations: DLPN, dorsolateral pontine nucleus; DMPN, dorsomedial pontine nucleus; INC, interstitial nucleus of Cajal; MT, middle temporal visual area; MST, medial superior temporal visual area; NRTP, nucleus reticularis tegmenti pontis; NPH, nucleus prepositus hypoglossi; VOR, vestibulo-ocular reflex. For further explanation, see text. **(A)** Modified from Fukushima (2003a,b) and Fukushima et al. (2006, 2011a).

Figure 5 depicts major pathways for smooth-pursuit (for reviews; see Lisberger et al., 1987; Robinson and Fuchs, 2001; Leigh and Zee, 2006). Briefly, the medial superior temporal (MST) cortical area is essential. From there, output signals are sent in two directions; one to pontine nuclei, primarily to the dorso-lateral pontine nuclei (DLPN), and through the cerebellar floccular region that includes the flocculus and ventral paraflocculus (Gerrits and Voogd, 1989), signals are sent to vestibular nuclei. The other direction is to the frontal cortex that includes the caudal part of the frontal eye fields (caudal FEF) and supplementary eye fields (SEF). From there, signals are sent to the nucleus reticularis tegmenti pontis (NRTP), and through the cerebellar dorsal vermis lobules VI–VII (i.e., oculomotor vermis) and its output region (i.e., the caudal fastigial nucleus, see Noda, 1991, for a review), signals are further sent to vestibular nuclei.

Output signals from the vestibular nuclei are sent directly, and also indirectly through the nucleus prepositus hypoglossi (NPH) or interstitial nucleus of Cajal (INC), to extraocular motoneurons (Figure 5A). These indirect pathways are involved in integration of eye velocity signals to eye position, common for all conjugate eye movements that consist of smooth-pursuit, saccades, optokinetic eye movements, and vestibulo-ocular reflex (VOR) (i.e., common neural integrator, Robinson, 1975; for a review, Fukushima et al., 1992). The cerebellar flocculus is also necessary for the neural integrator function (see Leigh and Zee, 2006 for a review). Signals in the cerebellar nuclei and vestibular nuclei are also sent to the cerebral cortex through the thalamus (Figure 5A, see Ito, 1984 for a review; also, Kyuhou and Kawaguchi, 1987; Noda et al., 1990; Fukushima, 1997).

Smooth-pursuit is required even when our head and/or whole body moves (for review see Barnes, 1993). Consistent with this requirement, vestibular-related signals induced by whole body rotation/translation, which activates semi-circular canals/otolith organs, are found in wide areas of the cerebral cortex including virtually all brain regions related to smooth-pursuit (Figure 5A; for reviews, Fukushima et al., 2011a; Goldberg et al., 2012; also Miyamoto et al., 2007; Schlindwein et al., 2008 for functional magnetic resonance imaging (fMRI) studies using high intensity clicks that selectively stimulate the sacculus).

### Memory-based smooth-pursuit

As noted earlier, efficient pursuit requires selection of the target to be pursued and predictive compensation for inherent delays in responses to target motion to ensure clear vision about the target. Prediction is influenced by various factors such as cues and working memory of stimulus trajectory (e.g., Badler and Heinen, 2006; Barnes and Collins, 2011; see Barnes, 2008, for review). Prediction could occur not only in motor commands to prepare for and maintain ongoing movements but also in the sensory and/or perception pathways (e.g., Barborica and Ferrera, 2003). Such mechanisms use memory (e.g., Assad and Maunsell, 1995; see section “Major Cognitive Influences on Pursuit Behavior”). However, our understanding of neural mechanisms of predictive pursuit is still incomplete.

Prediction-related neuronal discharge during smooth-pursuit was reported in the SEF (Heinen, 1995; Heinen and Liu, 1997; Kim et al., 2005; de Hemptinne et al., 2008) and caudal FEF (e.g., MacAvoy et al., 1991; Fukushima et al., 2002). Prediction-related activation of these areas during smooth-pursuit was also reported by fMRI in humans (e.g., Schmid et al., 2001; Burke and Barnes, 2008b). However, in these studies, activation related to preparation for pursuit eye movements could not be separated from activation related to processing of target motion signals or their working memory. Moreover, in daily life, a specific target must be selected from multiple moving objects, requiring decisions and selection of whether and what to pursue. Although the notion that the caudal FEF issues pursuit commands is well supported (MacAvoy et al., 1991; for a review, Fukushima et al., 2011a), the precise roles of the FEF and SEF in predictive pursuit were largely unknown.

**Figure 6:**
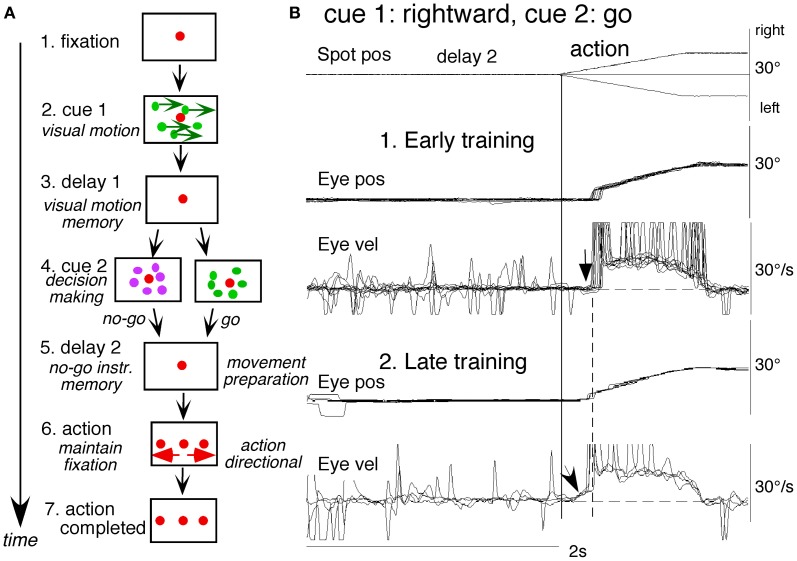
**A memory-based smooth-pursuit task and representative eye movements of a macaque monkey. (A)** Schematic illustration of the task. A red stationary spot appeared at the screen center and the monkeys were required to fixate it (**1**. fixation). Cue 1 consisted of a random-dot pattern of 10° diameter. All 150 dots moved along one of eight directions at 10°/s for 0.5 s [**2**. cue 1, 100% correlation of Newsome and Pare (1988)]. Visual motion-direction was randomly presented. The monkeys were required to remember both the color of the dots and their movement direction while fixating the stationary spot. After a delay (**3**. delay 1), a stationary random-dot pattern was presented as the 2nd cue for 0.5 s (**4**. cue 2). If the color of the stationary cue 2 dots was the same as the cue 1 color, it instructed the monkeys to prepare to pursue a spot that would move in the direction instructed by cue 1 (i.e., *go*). If the color of cue 2 differed from cue 1, it instructed the monkeys not to pursue (i.e., *no-go*) but to maintain fixation of a stationary spot which required remembering the *no-go* instruction during the 2nd delay (**5**. delay 2). *Go/no-go* cue was randomly presented. After the delay, the monkeys were required to execute the correct action by selecting one of three spots and either pursuing the correct spot in the correct direction or maintaining fixation (**6**. action). For this, the stationary spot remained centered, but spawned two identical spots; one that moved in the direction instructed by cue 1 and the other moved in the opposite direction at 10°/s. For correct performance, the monkeys were rewarded. For analysis, all trials were sorted by cue 1, cue 2 direction/instructions. **(B)** eye movement records during early and late training when cue 1 was rightward and cue 2 was *go*. Pos and vel indicate position and velocity. For further explanation, see text. Modified from Fukushima et al. (2008, 2011a) and Shichinohe et al. (2009).

To examine neuronal substrates for predictive pursuit, it is necessary to separate visual motion memory and movement preparation. For this, we employed a memory-based smooth-pursuit task that used two cues and two delay periods (Figure 6A; Shichinohe et al., 2009; Fukushima et al., 2011a,b): cue 1 indicated the visual motion-direction and cue 2 instructed whether to prepare to pursue (i.e., *go*) or not to pursue (i.e., *no-go*). Based on the memory of visual motion-direction presented at cue 1 (Figure 6A2) and the *go*/*no-go* instruction presented at cue 2 (Figure 6A4), monkeys were trained to decide which of two oppositely-directed targets should be selected and whether to pursue or not to pursue (by maintaining fixation of a third stationary spot) during the action period (**A6**, for further task explanation, see legend of Figure 6). This task thus invokes most of the mechanisms discussed in the section “Major Cognitive Influences on Pursuit Behavior.”

Figure 6B shows representative eye movement records of a representative monkey during early and late training when cue 1 was rightward and cue 2 was *go* (Fukushima et al., 2008, 2011a). Early in their training (typically after 6–8 months of training), monkeys learned the task basics with error rates of less than 10% for *go* and *no-go* trials. As illustrated in Figure 6B1, the monkey initiated the final action by saccades (but not by smooth-pursuit) with latencies typically 260–300 ms (**B1**, upward arrow), and these saccades were followed by smooth-pursuit. The lack of an initial smooth-pursuit component before saccades (**B1**, downward arrow) was consistent with the finding that vector averaging was used to combine visual inputs arising from two moving spots (Lisberger and Ferrera, 1997); in our task, visual motion inputs arising from the two oppositely moving spots with the same speed during the action period (Figure 6A6, e.g., leftward vs. rightward) would have been nullified (also Garbutt and Lisberger, 2006). Saccades to the cued direction during early training (Figure 6B1) must have enhanced visual motion processing of the pursuit target in that direction so that smooth-pursuit was effectively induced after saccades (i.e., postsaccadic enhancement of pursuit initiation, Lisberger, 1998; Ogawa and Fujita, 1998).

Later (typically after a year of training), saccade latency to spot motion shortened usually to about 220 ms, and preceding the saccades, initial smooth-pursuit appeared with latencies typically of 130–150 ms (Figure 6B2, arrow). This indicates that the acquisition of working memory and the appearance of the initial smooth-pursuit before saccades in this task are separate processes (Figures 6B1,B2; see section “Parkinson's Disease”); the latter required further training for efficient and nearly “automatic” tracking performance. Shortening of initial saccade latencies and appearance of the initial pursuit component in the late training are consistent with the interpretation that these responses were induced by priming effects of cue 1 direction memory and cue 2 *go* instruction (Bichot and Schall, 2002; Garbutt and Lisberger, 2006; see section “Representation of directional visual motion-memory and movement-preparation signals in the frontal cortex”).

### Neuronal activity in the major pathways related to smooth-pursuit

#### Representation of directional visual motion-memory and movement-preparation signals in the frontal cortex

**Figure 7:**
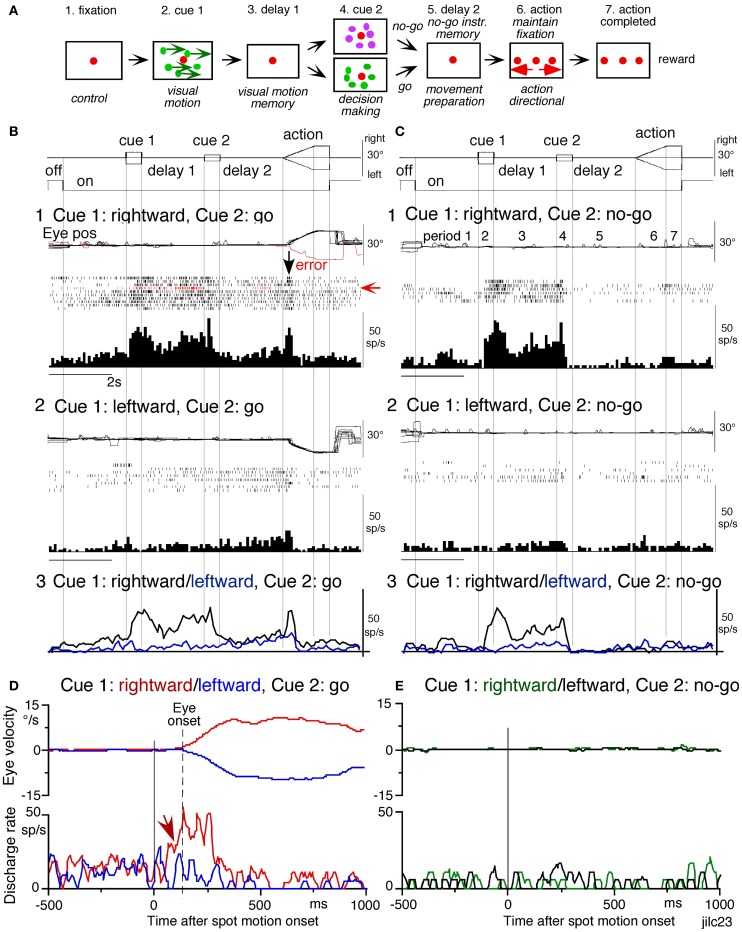
**Discharge of a representative SEF visual memory neuron. (A)** Task conditions. **(B1,2)**, *go* trials when rightward **(B1)** and leftward **(B2)** visual motion was applied as cue 1. **(C1,2)** *no-go* trials when rightward **(C1)** and leftward **(C2)** visual motion was applied as cue 1. Red trace in eye position (pos) record and arrow in spike raster in **(B1)** highlight an error trial. **(B3** and **C3)** compare mean discharge rate during rightward (black)/leftward (blue) cue 1 visual motion for *go* and *no-go* trials, respectively. To assess which period(s) of the task **(A2–7)** were associated with modulated neuronal activity, mean discharge rates of individual neurons were measured during the different task periods for the correct response [e.g., **(C1)**, periods 2–7], and were compared with the mean rate (±SD) during the initial fixation [**(C1)**, period 1] for each neuron. Significant differences were defined as those having a *p*-value < 0.05 using Student's t test with the Bonferroni correction for multiple comparisons. Neurons that exhibited significant modulation during this task were defined as task-related neurons. **(D** and **E)** de-saccaded and averaged eye velocity and discharge of this neuron 500 ms before and 1000 ms after spot motion onset (vertical straight line) during the action period. Smooth-pursuit onset is indicated by a dashed line. Only correct trials were averaged for *go* **(D)** and *no-go* conditions **(E)** as indicated by colors. See text for further explanation. Reproduced and modified from Shichinohe et al. (2009) and Fukushima et al. (2011a).

Using the memory-based smooth-pursuit task, signals for directional visual motion-memory and movement-preparation have been identified in the SEF and caudal FEF. Three groups of neurons were found; two of them carried these signals separately (visual memory neurons, movement-preparation neurons) and the third carried both signals (visual memory + movement-preparation neurons). Although the two regions carried qualitatively similar signals, consistent with the anatomical studies that show reciprocal connections between the SEF and FEF (Huerta et al., 1987), there were significant quantitative differences in the task-related signals represented in the two areas (see Figure 7 legend for the definition of task-related neurons). SEF visual memory neurons were unrelated to pursuit, whereas some FEF visual memory neurons were pursuit neurons (Shichinohe et al., 2009; Fukushima et al., 2011b).

***Visual memory neurons.*** Visual memory neurons exhibited direction-specific discharge during delay 1. An example SEF neuron (Figure 7) responded when rightward (but not leftward) visual motion was presented at cue 1; responses to cue 1 and during delay 1 were similar during *go* and *no-go* trials (**B1**,**B2** vs. **C1**,**C2**). The delay 1 discharge was not significantly influenced by the monkey's preparation of pursuit (**B1** vs. **C1**). This was also seen when the monkey erred (Figure 7B1, red trace in eye pos) by performing leftward (instead of rightward) pursuit. Despite this error, discharge similar to that during correct trials was clearly observed during delay 1 (**B1**, red raster). Moreover, it did not exhibit directional responses during delay 2 of *go* (**B3**, blue vs. black) or *no-go* trials (**C3**, blue vs. black). These results suggest that the delay 1 activity of visual memory neurons reflected memory of the visual motion-direction presented by cue 1. Although it exhibited a build-up activity during *go* trials (Figures 7B1,B2), it is unlikely that the activity was used directly for movement preparation, since it was non-directional (Figure 7B3).

Possible neural correlates for the putative priming effects by cues during the action period (Figure 6B2, arrow, section “Memory-Based Smooth-Pursuit”) are suggested in Figures 7B,C for this SEF visual memory neuron that had rightward preferred direction to cue 1 visual motion (**B1, C1**). Since this neuron was unrelated to pursuit (Figures 7B1,B2, action), the initial burst during the action period of *go* trials (**B1**, downward arrow) must have reflected visual response to rightward spot motion. Notice selective burst discharge to the identical visual motion stimuli during the action period, i.e., the clear burst during the action period appeared only in Figure 7B1 when cue 1 visual motion was rightward and cue 2 instruction was go (vs. **B2, C1**,**2**), indicating that the spot motion responses clearly depended on the visual motion-direction memory and *go*/*no-go* instructions. This interpretation is confirmed in Figure 7D; discharge to spot motion clearly occurred before the onset of the initial smooth eye velocity (D, red arrow before eye onset vs. other conditions D, E). Similar modulation of spot motion responses during the action period by cues was also observed in visual motion responses of some caudal FEF pursuit neurons (Figures 2F–I of Fukushima et al., 2011b).

**Figure 8:**
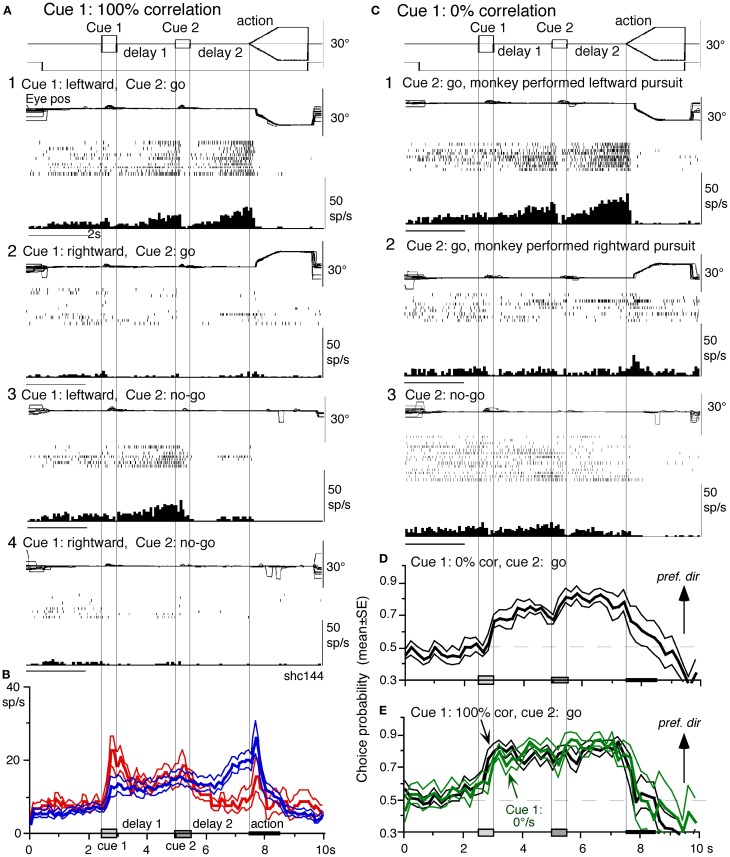
**Visual memory + movement-preparation neurons and comparison with visual memory neurons.** (**A** and **C**) Discharge of a representative SEF visual memory + movement-preparation neuron. Cue 1 motion-direction was presented as 100% correlation **(A)** and 0% correlation **(C)**. **(A1,2)** *go* trials when cue 1 motion was leftward **(A1)** and rightward **(A2)**. **(A3,4)** *no-go* trials when cue 1 motion was leftward **(A3)** and rightward **(A4)**. **(B)** Time course of mean (±SE) discharge modulation of visual memory neurons (red, *n* = 13) and visual memory + movement-preparation neurons (blue, *n* = 22) during *go* trials in their preferred directions. In **(C1,2)** *go* trials were sorted into leftward pursuit **(C1)** and rightward pursuit **(C2)** during action period. **(C3)** *No-go* trials. (**D** and **E**) plot mean (±SE) choice probability time course of 10 SEF visual memory + movement-preparation neurons during *go* trials based on whether the monkeys pursued in the preferred directions of individual neurons during delay 2 when cue 1 was presented with 0% correlation **(D)** and 100% correlation (**E**, black). Green traces in **(E)** are mean (±SE) choice probability time course of the same 10 neurons when a stationary pattern was presented at cue 1 (0°/s). For further explanation, see text. Reproduced and modified from Shichinohe et al. (2009) and Fukushima et al. (2011a).

***Visual memory + movement-preparation neurons.*** Visual memory + movement-preparation neurons exhibited direction-specific discharge during both delay 1 and delay 2. An example SEF neuron (Figures 8A1–A4) showed clear discharge during the late period of delay 1 when leftward visual motion was presented at cue 1 during *go* and *no-go* trials (**A1** vs. **A2, A3** vs. **A4**). In addition, when the cue 2 instructed *go* to prepare to pursue in the congruent direction (**A1**), it exhibited robust discharge during the late period of delay 2. Figure 8B plots a difference in time course of mean discharge of visual memory neurons (red) and visual memory + movement-preparation neurons (blue) in the SEF during *go* trials in their preferred directions. While the initial response to cue 1 for visual memory neurons (B, red) was larger, the two groups of neurons displayed similar discharge during the delay 1 and cue 2. During delay 2, the discharge of the two groups of neurons diverged.

Visual memory + movement-preparation neurons exhibited congruent directionality during delay 1 and delay 2 of *go* trials (Figures 8A1,B, blue). Our results suggest that the delay 1 information about the visual motion-direction is used for further processing in preparing for pursuit direction in the SEF (Shichinohe et al., 2009). This interpretation was examined in the following experiments. First, to examine how delay 1 and 2 responses were correlated, we let the monkeys choose the pursuit direction and examined how these neurons discharged during these periods. For this, we used the paradigm devised by Newsome and Pare (1988, 0% correlation) that moved each dot randomly in different directions at cue 1. In this condition, cue 1 does not provide the necessary information about the visual motion-direction. If the color of cue 2 was the same as cue 1, it instructed *go* and the monkey followed one of the two moving spots. If the color of cue 2 was different from that of cue 1, it instructed *no-go*, and the monkeys' maintained fixation. Each trial was sorted based on the monkeys' choice of either the preferred direction of delay 2 activity or the anti-preferred direction of the neuron (tested by 100% correlation).

Figure 8C plots sorted trials during 0% correlation for leftward pursuit (**C1**), rightward pursuit (**C2**) and *no-go* (**C3**) of the same neuron (A). When the monkey made leftward pursuit (i.e., in the preferred direction of this neuron at 100% correlation, Figure 8A), discharge during delay 2 was much stronger compared to the trials where the monkey made rightward pursuit (**C1** vs. **C2**), indicating that the delay 2 activity indeed reflected preparation for pursuit. In addition, the stronger discharge during the delay 1 in the same trials (**C1** vs. **C2**) suggests that this discharge during delay 1 was also related to the monkey's choice and preparation for the subsequent pursuit direction independent of the cue 1 stimulus itself, which was non-directional.

Second, to evaluate these results, we calculated choice probability (Britten et al., 1996) and its time course based on whether the monkeys pursued in the preferred direction of the neuron (tested by 100% correlation) or anti-preferred direction. The results for 10 SEF visual memory + movement-preparation neurons are plotted in Figure 8D. Mean choice probability values (which were ~0.5 before cue 1) increased above 0.7 during delay 1 and delay 2. For comparison, the time course of choice probability of the 10 neurons during 100% is plotted in Figure 8E (black). Also plotted in green (Figure 8E) was choice probability time course of the same 10 neurons when a stationary pattern (i.e., 0°/s) was presented at cue 1. The 3 curves (Figures 8D,E) were basically similar, indicating that delay 1 discharge was not a simple holding of visual motion response; the delay 1 response did not require visual motion stimuli, but reflected motion-direction assessment and memory (Fukushima et al., 2011a).

The congruent directionality of delay 1 and 2 discharge of visual memory + movement-preparation neurons was also observed when moving two spots stepwise during the action period so that the monkeys made saccades instead of smooth-pursuit (Shichinohe et al., 2009). These results suggest a common mechanism for visual memory and movement preparation for efficient tracking performance that includes both smooth-pursuit and saccades (Krauzlis, 2005).

#### Similarity and differences of signals represented in the SEF and caudal FEF

**Figure 9:**
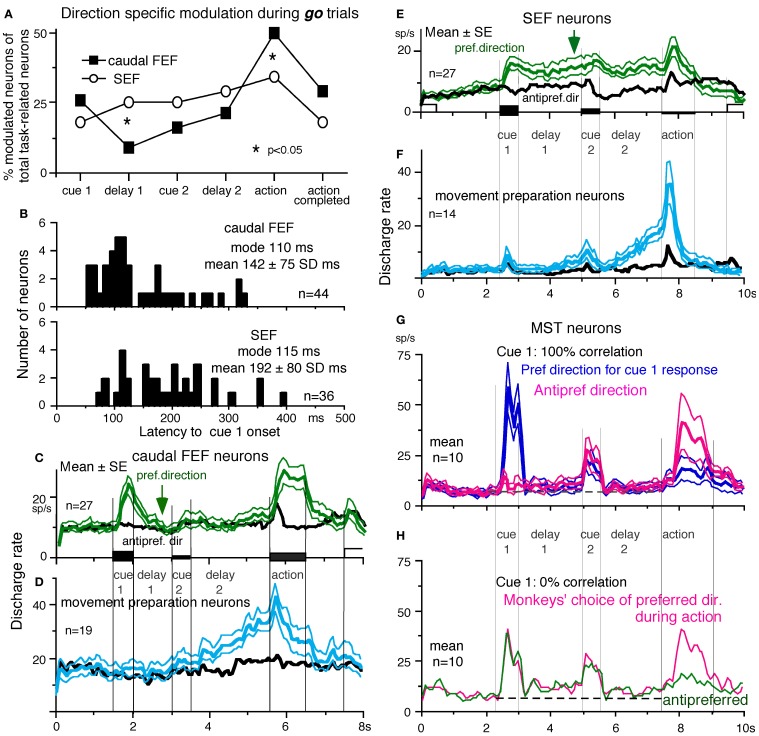
**Comparison of SEF and caudal FEF neuron discharge during memory-based pursuit. (A)** Comparison of percent of modulated neurons in caudal FEF and SEF (of total task-related neurons) that exhibited direction-specific modulation during *go* trials. See legend of Figure 7 for the definition of task-related neurons. **(B)** Comparison of latencies of visual motion responses of caudal FEF and SEF neurons to cue 1. Neurons with shorter visual latencies were significantly more frequent in caudal FEF than SEF (*p* < 0.01). **(C)** Mean±SE discharge of 27 caudal FEF neurons that exhibited directional visual motion response to cue 1 during *go* trials. **(E)** Mean±SE discharge of 27 SEF neurons that exhibited directional visual motion response to cue 1 during *go* trials. In (**C** and **E**) Green and black traces are discharge modulation in the preferred direction and anti-preferred direction, respectively. (**D** and **F**) Mean±SE discharge of movement- preparation neurons in the caudal FEF **(D)** and SEF **(F)** during *go* trials. Blue and black traces are discharge modulation in the preferred direction and anti-preferred direction, respectively. (**D** and **F**) Reproduced from Shichinohe et al. (2009). (**G** and **H**) Reproduced from Kurkin et al. (2011). Others, reproduced from Fukushima et al. (2011a,b).

To compare direction-specific discharge modulation during different task periods of *go* trials in the caudal FEF and SEF, Figure 9A plots the percent of modulated neurons (out of the total number of task-related neurons in each area) that showed direction-specific modulation in each period (e.g., Figure 7C1, periods 2–7). Although qualitatively similar signals were found in both areas, there were quantitatively significant differences between the two areas during delay 1 and action period (Figure 9A ^*^, Fukushima et al., 2011b); the percent of modulated neurons in the caudal FEF was significantly lower than that of the SEF during delay 1 but higher than that of the SEF during the action period. No significant difference between the two areas was detected in other periods including the delay 2 of *go* trials where movement-preparation is required.

FEF neurons exhibit visual latencies comparable with those in the middle temporal area (MT) and MST and sometimes even as early as some neurons in V1 (Schmolesky et al., 1998). Comparison of visual latencies of neurons that exhibited directional visual motion responses to cue 1 indicates that neurons with shorter visual latencies were significantly more frequent in the caudal FEF than the SEF (Figure 9B, Fukushima et al., 2011b). To examine how the difference between the two areas during delay 1 that signals directional visual motion-memory was reflected in the time course of mean discharge, Figure 9C plots discharge of caudal FEF neurons that exhibited directional responses to cue 1 in their preferred (green) and anti-preferred direction (black) during *go* trials. Although caudal FEF neurons exhibited a residual visual motion response to cue 1 that reflected directional visual motion-memory at the beginning of delay 1, the responses returned to control level near the end of delay 1 before cue 2 onset (Figure 9C, arrow). This contrasts with the discharge of SEF neurons that exhibited directional responses to cue 1 visual motion; cue 1 discharge was maintained during the whole delay 1 period (Figure 9E, arrow).

**Figure 10:**
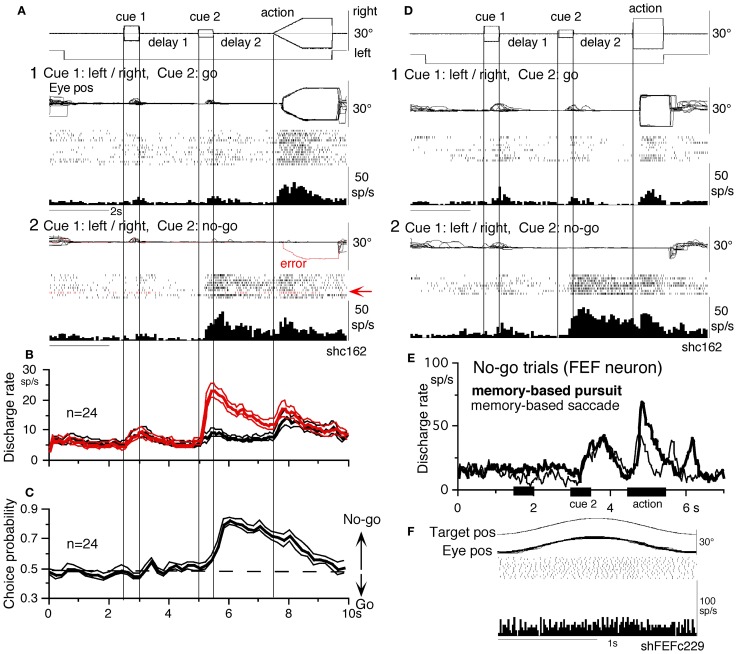
***No-go* neurons in the SEF and caudal FEF.** (**A** and **D**) A representative SEF *no-go* neuron during memory-based pursuit **(A)** and memory-based saccades **(D)**. **(A1)** *go* trials when rightward and leftward visual motion was applied as cue 1. **(A2)** *no-go* trials. Red trace in eye position record (arrow) and arrow in spike raster highlight an error trial. **(B)** Time course of mean (±SE) discharge of the 24 *no-go* SEF neurons during *no-go* (red) and *go* (black) trials. **(C)** Choice probability time course for the 24 SEF *no-go* neurons during *no-go* and *go* trials. (**D1** and **D2**) *Go* and *no-go* trials during memory-based saccades, respectively. (**E** and **F**) Discharge of a representative FEF *no-go* neuron during *no-go* trials of memory-based pursuit (**E**, thick) and memory-based saccades (**E**, thin). **(F)** Simple pursuit of a single spot that moved sinusoidally. **(A–D)** Reproduced from Shichinohe et al. (2009) and Fukushima et al. (2011a). **(E,F)** Reproduced from Fukushima et al. (2011a,b).

***No-go neurons.*** *No-go* neurons exhibited *no-go* instruction-specific discharge during delay 2 *no-go* trials (Shichinohe et al., 2009). The proportion of *no-go* neurons (of the total number of task-related neurons) was significantly higher in the SEF than caudal FEF (50/248 = 24% vs. 16/185 = 9%, Fukushima et al., 2011b). As shown in Figure 10A, this example *no-go* neuron in the SEF exhibited discharge during the action period of *go* trials, regardless of the pursuit direction (**A1**). When the cue 2 instruction was *no-go* (Figure 10A2), it exhibited a stronger discharge during cue 2 and delay 2. The difference in discharge modulation during these periods is clear in the mean discharge rates during *no-go* and *go* trials (Figure 10B, red vs. black). Furthermore, when the monkey erred during the action period of a *no-go* trial by pursuing a leftward moving spot (**A2**, red trace), this *no-go* neuron nearly stopped discharging at cue 2 and during delay 2, suggesting that the discharge during these periods reflected the monkey's decision not to pursue during *go* trials. This interpretation was supported by the analysis of choice probability (Britten et al., 1996; Zaksas and Pasternak, 2006) during delay 2 with respect to the monkeys' choice based on whether they maintained fixation (i.e., *no-go*) or if they pursued a moving spot, regardless of its directions (Figures 10A1 vs. A2). The choice probability increased to ~0.8 after cue 2 and decreased during the action period (Figure 10C). Latencies of *no-go* discharge relative to cue 2 onset were distributed widely with modal values of 160 ms for SEF and 180 ms for caudal FEF (Shichinohe et al., 2009; Fukushima et al., 2011b).

*No-go* related SEF discharge during delay 2 was also observed when monkeys performed memory-based saccades (Figures 10D1 vs. D2, Shichinohe et al., 2009). Discharge characteristics of *no-go* neurons in the caudal FEF were similar to SEF *no-go* neurons (Figure 10E), suggesting that *no-go* signals in SEF and caudal FEF were common during delay 2 that requires *no-go* instruction memory (Figure 6A5) for memory-based smooth-pursuit and saccades (Figures 10A,D,E, also Krauzlis, 2005). None of *no-go* neurons tested exhibited discharge modulation during simple pursuit using a single spot (Figure 10F), indicating that *no-go* neurons differ from pursuit neurons. *No-go* neurons are also different from fixation neurons in the FEF (Izawa et al., 2009, 2011), since *no-go* neurons in the memory-based pursuit/saccade task exhibited significant discharge only after cue 2 but not before (e.g., during cue 1 or delay 1), despite that the monkeys fixated a stationary spot during these periods (Figures 10A–E). *No-go* neurons were reported in a saccadic *go/no-go* task in the dorsomedial frontal cortex (Mann et al., 1988) and prefrontal cortex and FEF (Hasegawa et al., 2004).

***Movement-preparation neurons.*** Movement-preparation neurons exhibited direction-specific discharge during the delay 2 of *go* trials (Shichinohe et al., 2009). Figures 9D,F compare discharge modulation of movement-preparation neurons in the caudal FEF (D) and SEF (F); their time courses were similar. There was no significant difference in the percent of movement-preparation neurons (Figure 9A, delay 2) between the two areas.

#### Other cerebral cortical areas

Our knowledge of where the SEF visual memory signals are generated is still imprecise. The dorsolateral prefrontal cortex has been linked to temporal storage of sensory signals (i.e., working memory, Goldman-Rakic, 1995). Kim and Shadlen (1999) demonstrated that visual motion responses could be maintained during a delay period in prefrontal cortex neurons. However, in their studies, discharge related to the memory of visual motion could not be separated from discharge related to movement-preparation (also Zaksas and Pasternak, 2006).

Another potential site is MST, since this region, especially the dorsomedial MST (MSTd, Desimone and Ungerleider, 1986), sends direct projections to the SEF (Huerta and Kaas, 1990), and MSTd is involved in perception and memory of visual motion (e.g., Celebrini and Newsome, 1994; Britten and van Wezel, 2002; Gu et al., 2007; Liu and Angelaki, 2009; cf. Heuer and Britten, 2004). However, as illustrated in Figures 9G,H, representative signals in MSTd clearly differed from those in the SEF during memory-based smooth-pursuit; MSTd neurons signaled visual motion accurately, but none of the 108 MSTd neurons that showed directional visual motion response to cue 1 exhibited direction- and/or instruction-specific discharge during delay 1 or delay 2. Although they did show significantly higher discharge rates during the delay periods compared to the control period (Figures 9G,H, delay 1 and delay 2), their discharge was not directional (Kurkin et al., 2011), which suggests that their activity during the delay periods most probably reflected an effect of attention (e.g., Recanzone and Wurtz, 2000).

By manipulating visual inputs during pursuit eye movements, Newsome et al. (1988) demonstrated that the extraretinal, pursuit response of MSTd neurons begins at least 50 ms after onset of the smooth-pursuit eye movements, consistent with the behavioral findings of Barnes and Collins (2008a,b). They suggested that this response most likely derives from corollary discharge mechanisms and that MSTd plays a role in generating the motor signals responsible for the maintenance of ongoing pursuit. The results showing lack of movement preparation signals and late onset of MSTd neuron modulation during the action period of *go* trials (Figures 9G,H, Kurkin et al., 2011) are consistent with their observation (Newsome et al., 1988). The exact origin of the possible corollary discharge to MSTd is still unclear, but multiple brain areas including ventrolateral MST (MSTl, Thier and Erickson, 1992) seem to be involved. In particular, pursuit command signals issued from the caudal FEF could be sent directly to MST through cortico-cortical projections (Stanton et al., 1995) and also indirectly to MST via the descending pathways including the deep cerebellar nuclei and vestibular nuclei through the thalamus (Figure 5A, Schlag and Schlag-Rey, 1986; Tanaka, 2005; also Perrone and Krauzlis, 2008). Although we do not exclude possible alternative types of MSTd neurons coding assessment and memory of visual motion-direction (e.g., Ferrera and Lisberger, 1997), it seems more likely that visual motion-direction information sent from MSTd and caudal FEF to the SEF is further processed within the SEF to create assessment and the memory of visual motion-direction (Fukushima et al., 2011a,b; Kurkin et al., 2011).

#### Comparison of task-related discharge of the cerebellar oculomotor vermis/caudal fastigial nucleus and the floccular region

Signals similar to those seen in the SEF and caudal FEF were also represented in the oculomotor vermis/caudal fastigial nucleus and the floccular region, although clear differences were also observed (Fukushima et al., 2011c). In the floccular region, simple spike discharge of most task-related Purkinje cells responded only during the action period of *go* trials. None of them tested (41/44) exhibited significant modulation during delay 1 or 2 of *go* or *no-go* trials, suggesting that the floccular pathway is specifically involved in executing smooth-pursuit eye movements *per se* as reported earlier (Robinson and Fuchs, 2001; Leigh and Zee, 2006; Lisberger, 2009).

In contrast, most task-related Purkinje cells (50/76 = 66%) in the oculomotor vermis showed *no-go* instruction-specific discharge during cue 2 and delay 2 (Fukushima et al., 2011c). Their activity was not modulated during sinusoidal pursuit using a single spot, suggesting that it was unrelated to eye movement *per se*.

In our task, some task-related Purkinje cells (10/76) in the oculomotor vermis were pursuit-related during memory-based pursuit. Discharge characteristics of these neurons during pursuit using a single spot were similar to those reported previously (Robinson and Fuchs, 2001; Leigh and Zee, 2006); some of them also carried visual motion responses including memory and movement preparation-related discharge (Fukushima et al., 2011c).

In the caudal fastigial nuclei (cFN), the major response type (46/77 = 60%) was also *no-go* neurons (Fukushima et al., 2011c). Although neurons with discharge related to eye movement *per se* were in the minority in the memory-based pursuit task, some of them carried visual motion-memory and movement preparation signals. *No-go* neurons are different from omni-pause neurons in the brainstem that are active during fixation by suppressing burst neuron activity (see Leigh and Zee, 2006 for a review), since *no-go* neurons in the memory-based pursuit task exhibited significant discharge only after cue 2 but not before (e.g., during cue 1 or delay 1), similar to *no-go* neurons in the SEF and caudal FEF, despite that the monkeys fixated a stationary spot during these periods (Figure 10).

What do *no-go* neurons in the oculomotor vermis/cFN signal? We believe that *no-go* neurons in these regions are non-motor neurons that receive inputs from SEF/caudal FEF *no-go* neurons (Figure 5A) and signal *no-go* (i.e., not to pursue) memory during delay 2 for the following reasons. (1) Discharge characteristics of *no-go* neurons in all these areas were basically similar (Figures 10B,E), but mean latencies (re cue 2 onset) of *no-go* responses in the oculomotor vermis/cFN were significantly longer (>250 ms, *p* < 0.001) than those of SEF/caudal FEF *no-go* neurons (Fukushima et al., 2011c). (2) None of *no-go* neurons tested in the 4 areas exhibited directional eye movement-related discharge (e.g., Figure 10F). (3) Mean discharge rates of *no-go* neurons during the action period were similar during *go* and *no-go* trials (e.g., Figure 10B), consistent with the results showing that *no-go* neurons coded useful information during delay 2 (judged from choice probability with respect to the monkeys' choice for *no-go* or *go*), but choice probability quickly decreased during the action period. (4) During the action period of *no-go* trials, the monkeys occasionally made small saccades with amplitudes 1–6° (e.g., Figures 6A,B of Fukushima et al., 2011b). Discharge rates of *no-go* neurons during the delay 2 of such conditions were virtually identical to those when the monkeys fixated the stationary spot well without making such saccades (e.g., Figures 6A2 vs. B2 of Fukushima et al., 2011b), suggesting that their discharge during delay 2 was unrelated to the appearance/suppression of small saccades during the action period. We suggest that *no-go* neurons in these 4 areas may form part of cerebro-cerebellar network (Ito, 1984, Figure 5A) for *no-go* memory thereby they are involved in target selection.

In previous studies using conventional pursuit or saccade tasks, monkeys were not required to perform a *go/no-go* selection; *no-go* signals could not be identified. Possible involvement of the oculomotor vermis-caudal fastigial nucleus pathway in working memory for *no-go* instructions in monkeys may be a result of training (section “Memory-Based Smooth-Pursuit”) and part of cerebellar involvement in memory (see Ito, 2006, 2011, for reviews). Of note, Vastagh et al. (2005) examined the postnatal development of the Purkinje layer in the mouse cerebellum and showed that the oculomotor vermis belongs to the latest developing cerebellar cortical structures. Coffman et al. (2011) showed that the motor-related frontal cortical areas send massive projections to the cerebellar vermis including the oculomotor vermis. These observations indicate a close functional connection between the frontal cortex and the oculomotor vermis.

### Chemical inactivation

#### Different effects induced by chemical inactivation of the SEF and caudal FEF

Significant quantitative differences in signals represented in the two areas (sections “Representation of directional visual motion-memory and movement-preparation signals in the frontal cortex,” and “Similarity and differences of signals represented in the SEF and caudal FEF”) are consistent with the differences in the effects of chemical inactivation (Shichinohe et al., 2009; Fukushima et al., 2011b). Infusion of GABA agonist muscimol into the SEF resulted in significantly higher direction errors during *go* trials and *go/no-go* selection errors during *no-go* trials. Such errors were not induced by caudal FEF inactivation. Also, consistent with the existence of movement-preparation neurons in both areas (Figures 9D,F), chemical inactivation of either area impaired an initial smooth-pursuit component before saccades. Furthermore, since both areas contained neurons (visual memory neurons and pursuit neurons) that showed visual motion response enhancement to the cued spot during the action period (e.g., Figures 7B,D, arrows), loss of their activity may also have contributed to the impaired initial pursuit. In addition, consistent with the significant difference in percent of pursuit neurons in the two areas (Figure 9A, action), caudal FEF inactivation significantly decreased pursuit eye velocity during pursuit maintenance, resulting in saccadic tracking, whereas SEF inactivation did not impair pursuit maintenance. In particular, caudal FEF inactivation not only decreased eye velocity gain, but impaired delay compensation of pursuit eye movements during sinusoidal pursuit of a singe spot at frequencies ~1 Hz, suggesting that the caudal FEF is necessary for response delay compensation during sinusoidal pursuit.

These results indicate that the SEF is primarily involved in planning smooth-pursuit, whereas the caudal FEF is primarily involved in generating motor commands for pursuit execution. The existence of *no-go* neurons along with impairment in performing *no-go* trials after chemical inactivation suggests that the SEF is necessary for decision-process of whether or not to pursue moving spots including working memory of *no-go* instructions (Shichinohe et al., 2009; Fukushima et al., 2011a,b).

After inactivation of either area, postsaccadic enhancement of smooth-pursuit (Lisberger, 1998) was still observed (Shichinohe et al., 2009; Fukushima et al., 2011a,b), indicating involvement of different neural mechanisms in generating the initial pursuit component and postsaccadic pursuit enhancement. Mahaffy and Krauzlis (2011) reported that inactivation and stimulation of the frontal pursuit area change pursuit metrics without affecting pursuit target selection, consistent with our muscimol inactivation of the caudal FEF (Fukushima et al., 2011b).

#### Chemical inactivation of the caudal fastigial nucleus

Unilateral chemical inactivation of the caudal fastigial nucleus induces well-known impairments in smooth-pursuit and saccades (e.g., dysmetria, for reviews, see Robinson and Fuchs, 2001; Leigh and Zee, 2006). In addition, during memory-based smooth-pursuit, chemical inactivation of the caudal fastigial nucleus induced significantly higher *no-go* errors as well as direction errors (mean 40 vs. <10% before inactivation, Fukushima et al., 2011c), indicating impairments of visual working memory in this task. These results suggest that the oculomotor vermis-caudal fastigial nucleus pathway is involved in planning tracking eye movements that includes both smooth-pursuit and saccades, similar to the SEF (Shichinohe et al., 2009).

## Preliminary results of clinical application

### Parkinson's disease

Characteristic of Parkinson's disease (PD) are difficulties in initiating volitional movements and, when initiated, slow and hypo-metric movement (e.g., Warabi et al., 2011). Ocular pursuit is impaired in most patients with PD, though the nature of the impairment is poorly understood (Leigh and Zee, 2006). Working memory impairment during cognitive tasks has been reported (e.g., Possin et al., 2008; Lee et al., 2010). To examine whether working memory of visual motion direction is impaired, we applied the memory-based smooth-pursuit task to patients with PD. None of the PD patients tested exhibited impaired working memory of motion-direction and/or *go/no-go* selection, indicating that these functions were normal in PD patients tested (Fukushima et al., 2011c), consistent with studies showing normal predictive function, including timing function, of most PD patients during smooth-pursuit (e.g., Waterston et al., 1996; Lekwuwa et al., 1999; also Pinkhardt et al., 2009; de Hemptinne et al., 2013).

**Figure 11:**
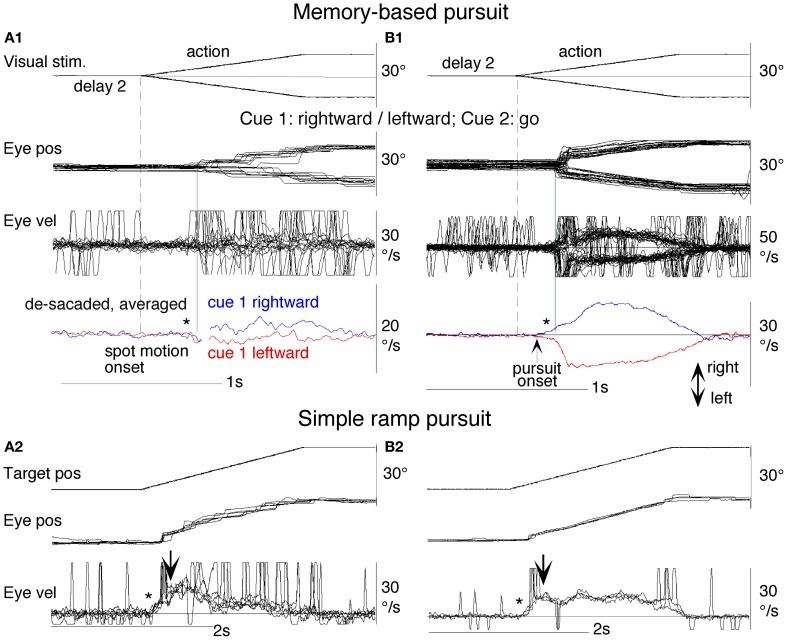
**Eye movements of a patient with Parkinson's disease and a normal control.** (**A1** and **A2**) Memory-based pursuit **(A1)** and simple ramp pursuit using a single spot **(A2)** of a Hoehn–Yahr stage III patient (73 years old). (**B1** and **B2**) A normal control during memory-based pursuit **(B1)** and simple ramp pursuit **(B2)**. In (**A1** and **B1**) *Go* trials with rightward and leftward cue 1 motion were combined, since both subjects made no errors. Eye velocity (vel) during saccades was clipped. Bottom traces in (**A1** and **B1**) are de-saccaded, averaged eye velocity for cue 1 rightward visual motion (blue) and cue 1 leftward (red) as indicated. Horizontal straight lines on eye velocity traces indicate zero velocity. ^*^Indicates presence or absence of the initial pursuit component. See text for further explanation. Reproduced from Fukushima et al. (2011a,c).

Clear differences from normal controls were observed during *go* trials. Normal controls exhibited initial smooth-pursuit component in the cued direction with a mean latency of 155 ms (Figure 11B1 ^*^) followed by corrective saccades (Fukushima et al., 2011a,c; cf. Garbutt and Lisberger, 2006) which were further followed by enhanced smooth-pursuit responses (cf. Figure 6B2; Lisberger, 1998). Note that this pattern of tracking eye movements is basically similar to the pattern observed in monkeys after late training of this task (see Figure 6B2). In contrast, most PD patients tracked the correct spot with saccades; initial pursuit was rarely induced before the saccades (Figure 11A1 ^*^), and postsaccadic enhancement of smooth-pursuit was rarely observed. Moreover, consistent with many previous reports, peak pursuit eye velocities after saccades were significantly lower (i.e., low gain) in PD patients than those of controls during pursuit maintenance (Figures 11A1 vs. B1, de-saccaded, averaged).

The lack of initial pursuit and deficient postsaccadic enhancement in most PD patients are unlikely to be due to impairments of smooth-pursuit eye movements *per se*, since during simple ramp pursuit of a single spot moving at the same velocity, the same patients clearly exhibited an initial pursuit component before saccades, similar to normal controls (Figures 11A2 vs. B2 ^*^), and since postsaccadic enhancement of smooth-pursuit was also seen at least for the first saccades after spot motion (**A2** and **B2**, downward arrows).

The appearance of the initial pursuit during the action period of memory-based pursuit in control subjects (Figure 11B1) most probably reflects priming effects by cues and depends on normal activity of the SEF and caudal FEF (sections “Representation of directional visual motion-memory and movement-preparation signals in the frontal cortex,” “Similarity and differences of signals represented in the SEF and caudal FEF,” Fukushima et al., 2011a), since in monkey studies cue 1 direction memory and cue 2 *go* instruction enhance visual motion responses of SEF and caudal FEF neurons in the cued direction (e.g., Figures 7B,D), and since chemical inactivation of these frontal cortical areas impairs initial pursuit before saccades (Shichinohe et al., 2009; Fukushima et al., 2011a,b).

Conversely, the lack of initial pursuit in patients with PD suggests that they have difficulty in inducing priming effects during memory-based pursuit (Figures 11A1 vs. B1 ^*^) which required the patients to prepare and execute smooth-pursuit to a selected spot using the cue information (Fukushima et al., 2011a; cf. Ladda et al., 2008).

Cui et al. (2003) reported projection of the FEF pursuit area to the basal ganglia (BG) in monkeys, output of which further project back to the caudal FEF through the thalamus, forming a possible efference copy loop between the caudal FEF and BG (Figure 5B, also see Tian and Lynch, 1996; Lynch and Tian, 2006). Yoshida and Tanaka (2009) suggested that this pursuit loop may contribute to maintaining normal pursuit gain (see also Basso et al., 2005). Our results suggest that, of the two major components of predictive pursuit, the visual motion-direction memory is normal but movement preparation is impaired in PD in addition to impaired movement execution. A common pathophysiology may contribute to low gain pursuit and hypokinesia (Warabi et al., 2012).

In contrast to normal working memory during memory-based pursuit in patients with PD, significantly higher error rates were observed in patients with frontal cortical dysfunction using the identical task; these patients revealed low perfusional volume in the frontal or frontotemporal cortex using single photon emission computed tomography (Ito et al., 2011). Dramatic impairment of prediction due to frontal lobe degeneration has also been reported by Coppe et al. (2012). These results suggest that PD patients with working memory impairment may have frontal cortical dysfunction that includes the SEF (e.g., Possin et al., 2008; Lee et al., 2010).

### Cerebellar degeneration

**Figure 12:**
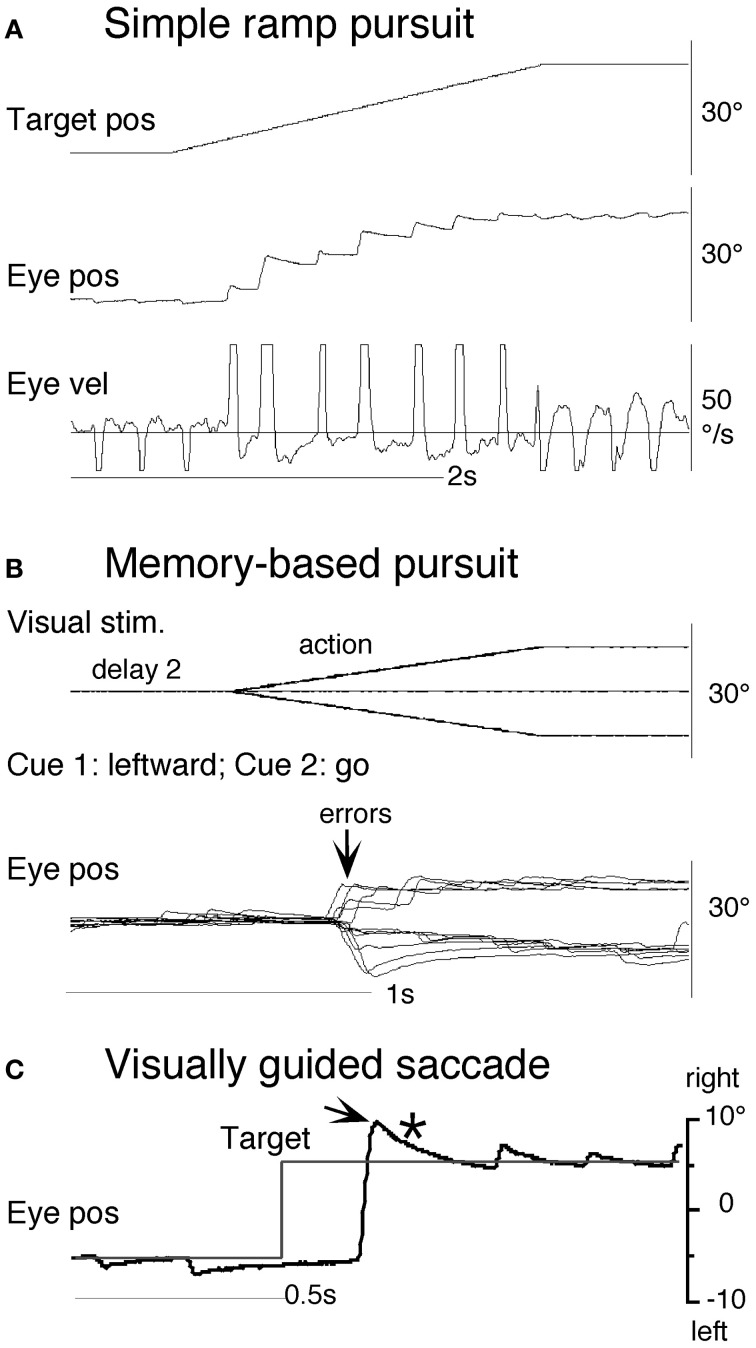
**Eye movements of a patient with spino-cerebellar degeneration. (A)** Simple ramp pursuit. **(B)** Memory-based pursuit when cue 1 visual motion was leftward and cue 2 instruction was go. **(C)** Visually guided saccade. Horizontal straight line on eye velocity trace in **(A)** indicates zero velocity. See text for further explanation. Reproduced and modified from Fukushima et al. (2012).

Most cerebellar patients exhibit well-known impairments of eye position holding failure due to impairment of the neural integrator (section “Major Pathways Related to Smooth-Pursuit Eye Movements,” Robinson, 1975; Leigh and Zee, 2006). As illustrated in Figure 12C1, a representative patient with cerebellar degeneration tracked a moving target with saccades. But unlike PD patients (e.g., Figure 11A2), corrective saccades of the cerebellar patient were followed by centripetal drift due to neural integrator failure, resulting in little pursuit eye velocity (Figure 12A; also Westheimer and Blair, 1973, 1974). Moreover, during visually guided saccades, the same patient exhibited dysmetric saccade (Figure 12C, arrow) that was followed by eye position holding failure (Figure 12C ^*^), suggesting that both the cerebellar floccular region and oculomotor vermis were dysfunctional. In addition, during memory-based pursuit, most cerebellar patients tested exhibited direction errors during the action period (Figure 12B), suggesting impaired visual working memory in this task as well (Fukushima et al., 2012). These differences between patients with PD and those with cerebellar degeneration (Figures 11 vs. 12) suggest different roles for the BG and cerebellum in smooth-pursuit planning and execution (cf., Allen and Tsukahara, 1974).

## Functional considerations

### Comparison of memory-based and simple ramp pursuit

**Figure 13:**
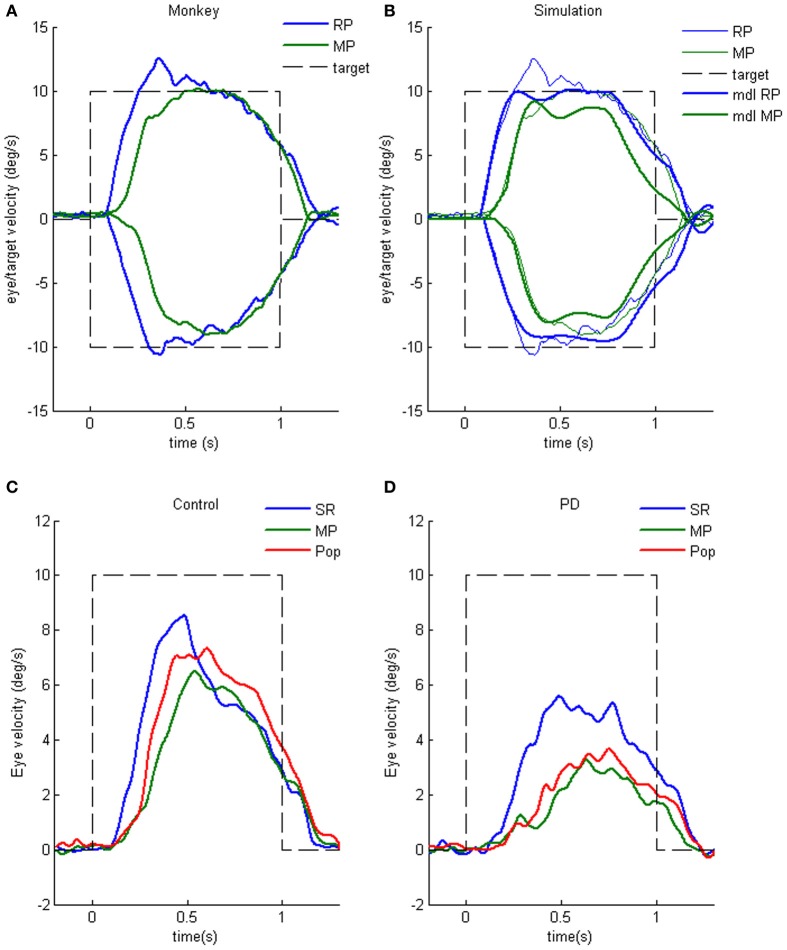
**Simple ramp and memory-based pursuit responses. (A)** Eye velocity responses to left (negative) and right (positive) from a single monkey during simple ramp pursuit (SR) vs. memory pursuit (MP). **(B)** Simulations of model (thick lines) corresponding to SR and MP responses (thin lines) shown in **(A)**. Parameter values (see Figure 2): Te = 0.12 s; β = 1; K_0_ = 3 (right); K_0_ = 2 (left). (**C** and **D**) Mean SR and MP responses compared with responses to memory pursuit with Popout (Pop) in six Controls and seven PD patients. Averages of left- and right-going responses. From Ito et al. (2012).

**Figure 14:**
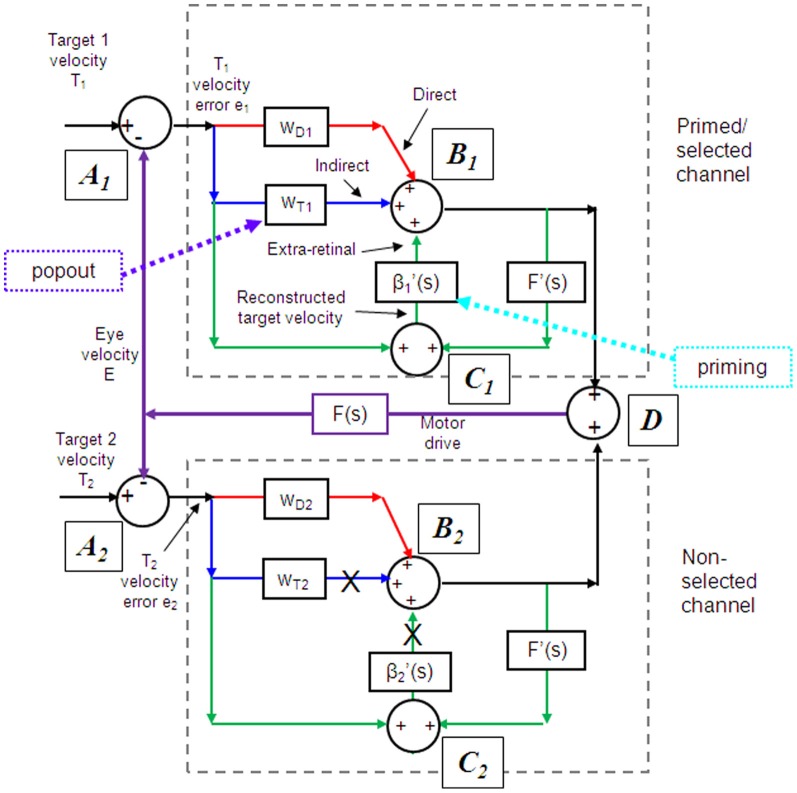
**Two channel model of pursuit.** Model of interactions between two oppositely directed targets via 2 SEF neurons with opposing preferred directions. Extra-retinal pathway components [S/H, MEM, β, and F”(s)] of Figure 2 have been reduced to a single function β′(s) and the main feedforward pathway has been split into direct (MST-DLPN) and indirect (MST-FEF-NRTP) components consistent with established pathways from MST to brainstem. Open-loop gain functions (equivalent to K in Figure 2) for direct and indirect pathways are represented as weighting factors (w_D_ and w_T_, respectively). Prior display motion primes the extraretinal pathway by increasing β′_1_ and by increasing w_T1_ once target selection occurs. Popout enhances and advances target selection. In the non-selected channel there is no priming of β′_2_ or w_T2_ which remain inactive as indicated by crosses. Adapted from Schweigart et al. (2003).

Although smooth pursuit is evoked in both monkeys and humans in the memory-based task, comparison with simple ramp responses reveals clear differences. Memory-based eye acceleration starts slightly later and is considerably less than in the simple ramp, but a transition to higher acceleration occurs 250–300 ms after target onset [Figures 13A (monkey), C (human)]. These differences probably result from competition between the dual identical targets in the memory pursuit task, which move in opposing directions and are continuously visible throughout the task (Lisberger and Ferrera, 1997). The interactions can be represented by the model in Figure 14 (adapted from Schweigart et al., 2003) in which the two channels correspond to neuronal structures with directional sensitivities of opposite polarity, similar to those shown in Figure 7. Retinal error input from each of the two targets interacts at junction D to create the final motor drive. Note that the extra-retinal pathway components [S/H, MEM, β, and F”(s)] of Figure 2 have been reduced to a single function β′(s) and the main feedforward pathway has been split into direct (MST-DLPN) and indirect (MST-FEF-NRTP) components, consistent with established pathways from MST to brainstem.

Our hypothesis is that active pursuit of a single target in the simple ramp task is achieved by augmentation of gain for the selected target by increasing open-loop gain (w_T1_) in the indirect pathway and concomitantly initiating extra-retinal activity in the efference copy loop (i.e., increasing β_1_). Raising gain in the indirect pathway (w_T1_) is the primary factor responsible for the initial high acceleration pursuit response, the extra-retinal component giving a lower level of eye acceleration and developing later than the visually driven component (see Figure 4D). By contrast, in the memorized pursuit task, priming by the prior display motion presentation (cue 1) facilitates activation of the extra-retinal component (i.e., β_1_ ≈ 1) in the appropriate channel but does not allow open-loop gain (w_T1_) to be immediately increased, thus leading to a low initial acceleration. Prior to initiation of the extra-retinal component weightings w_D1_ and w_D2_ are assumed to be equal and thus to cancel each other as a result of vector averaging (Ferrera and Lisberger, 1997); individually they would give a low-gain response of the type induced by passive stimulation (Cheng and Outerbridge, 1975; Barnes and Hill, 1984; Pola and Wyatt, 1985). We suggest that w_T1_ remains low because of difficulty in discriminating between the two identical, but oppositely directed, targets. When selection does occur, there is an abrupt increase in w_T1_ leading to a rapid increase in eye acceleration comparable to that seen in the simple ramp task. Model simulations of simple ramp and memory-based responses in the monkey are shown in Figure 13B. To assist discrimination we investigated the effect of stimulus popout by making the target in the cued direction change color at motion onset. This allowed abrupt acceleration to occur earlier (Figure 13C), an effect we attribute to an earlier increase in w_T1_.

Crucially, PD patients may not be capable of this modification of w_T1_ since their responses in the memory pursuit task do not show an abrupt increase in acceleration (Ito et al., 2012), even with a popout stimulus (Figure 13D). In addition, eye acceleration and peak velocity in the simple ramp task were lower than in Controls, consistent with previous observations of reduced gain in both anticipatory and visually-driven components of pursuit (Lekwuwa et al., 1999; Helmchen et al., 2012).

Notably, the initial low acceleration component of the memory-based response, which we attribute to the extra-retinal component, is absent in early training in the monkey, implying that it takes some time to train the animal to develop and release the extra-retinal response. This may be similar to a process described previously in the development of pursuit in juvenile monkeys (Shichinohe et al., 2011). Juvenile animals initially exhibit considerable instability that gradually disappears with practice. It was suggested that this could be explained by the gradual development of the extra-retinal component of pursuit. It is clear that there is a major species difference in the development of anticipatory movements and the extra-retinal component, since humans need only a few trials to learn how to generate such responses.

### Allocation of model functions to specific brain areas

Given the findings reported here and those of earlier experiments it is possible to tentatively allocate some of the functions of the behavioral models (Figures 2, 14) to specific brain areas. The reconstruction of target velocity at junction C in the models, which forms the basis of the extra-retinal component, is almost certainly carried out in MST/V5. It has long been assumed that MST plays an important role in the integration of retinal error and efference copy signals because of the sustained firing observed during target occlusion and image stabilization (Newsome et al., 1988). However, we have also taken into account the experimental results of Ilg et al. (2004) and the adaptive modeling of Dicke and Thier (1999), providing evidence that MSTl is an area in which not only retinal error and eye velocity, but also head velocity are integrated to provide an estimate of target velocity in world-centered coordinates, consistent with the modeling of results from head-free pursuit experiments (Ackerley and Barnes, 2011). In order to make internal target reconstruction temporally appropriate it is necessary to incorporate a delay in the efference copy feedback, so that if the inputs to junction C (Figure 2) are examined when operating in the reactive mode they comprise a retinal error signal and a delayed eye velocity efference copy signal, as observed in neuronal recordings (Newsome et al., 1988). However, if the system is operating in the predictive mode, initiating anticipatory eye movement on the basis of motion information previously stored in MEM, the activity in MST will be phase advanced with respect to that in the reactive mode; some evidence to support this has come from neuronal recordings in the monkey (Ilg, 2003).

Time-advanced neuronal activity has also been observed in FEF and SEF (Fukushima et al., 2002) during predictive pursuit of sinusoidal target motion. It is well-established that MST is connected bi-directionally with FEF (Huerta and Kaas, 1990) and an MST→FEF→MST feedback system might be one plausible way for the efference copy loop to operate, as outlined earlier. However, our results suggest MST may not be a velocity memory site *per se*, since no continued firing was observed during the delay periods of the memory pursuit task (Figure 9G). Whilst it is possible that such activity may be found in other parts of MST (e.g. MSTl), sustained firing here may, in fact, be dependent on ongoing eye movement. Given the evidence presented in section “Evidence of Sampling and Storage in the Initial Pursuit Response,” that velocity information may be sampled, an intact MST-FEF feedback loop is unlikely to be necessary for memory maintenance. Rather, it is likely that the velocity sample is held in a form of working memory, most probably in dorsolateral PFC (Schmid et al., 2001; Burke and Barnes, 2008b), which is a likely indirect recipient of MT and/or MST output (Kim and Shadlen, 1999; Barborica and Ferrera, 2003; Zaksas and Pasternak, 2006). Such an area may be responsible for holding sampled velocity information (i.e., to be the substrate for S/H and MEM) in a similar way to that for spatial information in remembered saccade tasks (Funahashi et al., 1990). Unlike the remembered target location in the saccade task though, the sample would be held as a magnitude (firing rate) estimate. Some behavioral evidence suggests that magnitude may indeed be stored irrespective of intended direction, since appropriately scaled anticipatory movements can be re-directed even without prior exposure to motion in the new direction (Jarrett and Barnes, 2002).

SEF is probably the area where decisions about the release of the extra-retinal component are controlled and, given the results presented in section “Similarity and differences of signals represented in the SEF and caudal FEF,” FEF is also likely to be involved in that process as a result of reciprocal interconnections with SEF (Huerta et al., 1987). The results of the memory-based pursuit task demonstrate first that, in visual memory neurons, there is sustained activity during the delay periods that is specific to the direction of the initial cue. It is likely that this sustained activity emanates from the working memory holding sampled motion information, although whether this information has speed as well as directional content is unknown (see section “Representation of directional visual motion-memory and movement-preparation signals in the frontal cortex”). It is clear from the fact that directional errors occur that the sustained activity in delay 1 is not irrevocably associated with a motor response in that direction or, indeed, with any motor response at all in the no-go condition. The implication is that an erroneous higher-level decision is made to follow the target in the non-primed direction or, in the case of the no-go condition, to suppress all response. A second subset of SEF and FEF neurons exhibit motor preparation activity in the form of steadily increasing firing rate prior to the motor response. This type of activity has been observed before in SEF (Heinen and Liu, 1997) and is known to be increased by increasing stimulus predictability. This preparatory activity appears to be linked to anticipatory smooth pursuit, which is also dependent on stimulus predictability (Heinen et al., 2005; de Hemptinne et al., 2007, 2008). Anticipatory eye movements are enhanced by stimulation in SEF (Missal and Heinen, 2004), probably through augmentation of this preparatory signal. At present this function is represented in the model by the modifiable gain component β, although this is a considerable simplification of a complex probability-dependent process.

SEF is also implicated in other decision making processes, notably the timing of response initiation and termination (Heinen and Liu, 1997) and may thus be a component of the timing mechanisms associated with the release of predictive activity (Figure 2) for which there is ample behavioral evidence (Barnes and Asselman, 1991; Jarrett and Barnes, 2005; Badler and Heinen, 2006). Storage of timing information is an important aspect of other motor control processes (see Ivry and Spencer, 2004, for review). Notably, SEF contains only a small proportion of neurons whose activity is directly related to the motor response (Fukushima et al., 2004); consistent with this, chemical inactivation of SEF (with intact FEF) does not impair pursuit maintenance (Shichinohe et al., 2009). We suggest, therefore, that the major role of SEF lies not in the direct transmission of motor activity but in the regulation of such activity between visual motion memory sites (S/H and MEM in the model) and FEF, which is the major output center for pursuit. This includes the important ability to control suppression of the motor output in the no-go condition.

FEF is probably the site at which retinal error and internal drive (either *reactive* or *predictive*) signals are summated (junction B in Figure 2), since lesions of the FEF are known to impair both predictive and visually guided components of smooth pursuit (Keating, 1991, 1993). As shown in Figure 9, many FEF neurons fire continuously throughout the action period in a way that would be expected at the output of this summing junction (see Figure 4F). However, another type of FEF output neuron that exhibits temporal characteristics more consistent with the visually driven (retinal error) component has also been identified (Fukushima et al., 2000; Ono and Mustari, 2009). It is possible, therefore, that this summation may take place further downstream in, for example, the vestibular nuclei.

The control of open-loop gain is another function frequently associated with FEF. Tanaka and Lisberger (2001) showed that microstimulation in FEF can enhance the gain of pursuit and Churchland and Lisberger (2005) have suggested that MST may be the site that controls gain via its links to FEF, consistent with the representation in Figure 2. Given the reduced gain in PD patients, an FEF→BG→FEF positive feedback loop may carry out this function (see section “Parkinson's Disease”).

### Implications for performance assessment in clinical disorders

Observation of reduced pursuit performance is common in patients with various neurological conditions, such as cerebral cortical lesions, cerebellar degeneration, PD, and schizophrenia (Leigh and Zee, 2006), so standard pursuit tasks offer little potential for differential diagnosis. What we demonstrate here is that suitably devised tests that take into account a fuller range of facets of pursuit may provide much more information. For example, the effects of chemical inactivation of FEF (Fukushima et al., 2011a,b) suggest an association between timing and velocity of the memory-based pursuit response and the gain and phase error of sinusoidal pursuit. Such effects have been observed before in patients with cortical lesions (Lekwuwa and Barnes, 1996a,b) but localization has proved difficult because the tasks used did not clearly discriminate between factors such as gain control, timing and expectation. By continuing to investigate neuronal activity with more elaborate memory-based pursuit tasks that improve discrimination by adding factors such as storage of speed information, it should be possible to identify more areas that are critical for particular factors.

### Conflict of interest statement

The authors declare that the research was conducted in the absence of any commercial or financial relationships that could be construed as a potential conflict of interest.
